# Traditional Chinese Medicine Injections Combined With Oseltamivir for Influenza: Systematic Review and Network Meta-Analysis

**DOI:** 10.3389/fphar.2022.848770

**Published:** 2022-07-22

**Authors:** Yingying Peng, Zhe Chen, Huanmin Li, Yaowei Han, Dan Sun, Yanjiao Li, Xiaoxia Wu, Hongxiang Chen, Xinmin Li

**Affiliations:** ^1^ First Teaching Hospital of Tianjin University of Traditional Chinese Medicine, Tianjin, China; ^2^ National Clinical Research Center for Chinese Medicine Acupuncture and Moxibustion, Tianjin, China; ^3^ Evidence-Based Medicine Center, Tianjin University of Traditional Chinese Medicine, Tianjin, China

**Keywords:** traditional Chinese medicine injections, oseltamivir, systematic review, network meta-analysis, clinical evidence, influenza, antiviral drug in Chinese herbal medicine

## Abstract

**Background:** As a cause of respiratory tract infections in humans, influenza remains with high morbidity and mortality, with associated significant healthcare burden and increased financial burden. Traditional Chinese medicine injections (TCMIs) combined with oseltamivir (TCMIs + oseltamivir) are the representative therapeutic strategies for influenza, which is a compliant with clinical applications in China. The aim of this study was to describe the comparative efficacy and safety of TCMIs + oseltamivir in patients with influenza, based on the current evidence.

**Methods:** PubMed, Embase, Cochrane Library, Web of Science, China National Knowledge Infrastructure, Wanfang Data Knowledge Service Platform, VIP information resource integration service platform databases, and the Chinese biomedical literature service system were searched to find randomized controlled trials where TCMIs + oseltamivir are the representative therapeutic strategies for influenza, from inception until October 2021, without language restriction. Two investigators independently screened eligibility criteria, extracted data, and appraised the risk of bias with the same criteria. We conducted a network meta-analysis using the Bayesian random method for each outcome and performed the sensitivity analysis, meta-regression, and Egger’s and Begg’s tests for the reliability and robustness of our results.

**Results:** Thirty-one trials including 2,893 participants proved eligible and reported on four TCMIs + oseltamivir *versus* oseltamivir. Network meta-analysis showed Yanhuning (YHN) +oseltamivir (MD = −1.7, 95% CrI: −2.5 to −0.88; SUCRA = 0.89; low certainty of evidence) in fever disappearance time, Tanreqing (TRQ) +oseltamivir (MD = −1.9, 95% CrI: −2.8 to −1; SUCRA = 0.97; low certainty of evidence) in cough disappearance time, and Xiyanping (XYP) +oseltamivir (OR = 5.9, 95% CrI: 3.1 to 11; SUCRA = 0.82; very low certainty of evidence) in the response rate to be more efficacious than oseltamivir alone with the best SUCRA. Based on the combined SUCRA value for primary outcomes, TRQ + oseltamivir is probably better in cough disappearance time, and XYP + oseltamivir and YHN + oseltamivir may be better in fever disappearance time than others. No significant difference in safety between the treatments.

**Conclusion:** In patients with influenza, TCMIs + oseltamivir only partially improve flu symptoms. Overall therapeutic efficacy and safety are inconclusive, based on low to very low certainty of evidence. However, the safety remains uncertain, and TCMI treatments for influenza should be considered with caution. More high-quality studies examining the efficacy and safety of TCMIs are needed.

**Systematic Review Registration:** https://www.crd.york.ac.uk/PROSPERO/, identifier CRD42021286994

## 1 Introduction

Influenza is one of the most prevalent causes of respiratory tract infections, which remains a public threat to health-related quality of life worldwide and further intensifies the considerable clinical and socioeconomic burden (Gilbert, 2018). Influenza viruses result in not only a series of symptoms (e.g., fever, headache, cough, nasal congestion, muscle soreness, and fatigue) in acute episodes of respiratory disease (Ghebrehewet et al., 2016; Uyeki 2017) but also gastrointestinal symptoms such as nausea and vomiting (Minodier et al., 2015). Influenza virus infections were associated with high morbidity and mortality worldwide each year, and its true mortality rate was higher than that reported by the World Health Organization (Simonsen et al., 2013; Coates et al., 2015). It is noteworthy that influenza is a leading cause of hospitalizations in pulmonary respiratory diseases, and it remains a significant health burden for the elderly, neonatal, and children (Thompson et al., 2004; Lafond et al., 2016; Cromer, 2014). Also, influenza viruses can contribute to the risk of secondary non-respiratory diseases, particularly in the cardiovascular and nervous systems (Sellers et al., 2017).

Neuraminidase inhibitors are still globally approved as currently used antiviral therapeutic agents for influenza, mainly including oseltamivir and zanamivir, and it is recommended to intervene early in the development of disease (Kumar 2011; Muthuri et al., 2014). In clinical practice, oseltamivir is a powerful antiviral drug widely used to treat influenza A and B infections (Davies, 2010). Oseltamivir has been demonstrated to alleviate symptoms such as cough, headache, nasal congestion, and fatigue, and reduce other respiratory complications and hospitalization risks, as well as the mortality of hospitalized patients with influenza (Jefferson et al., 2014; Muthuri et al., 2014; Dobson et al., 2015). With the widespread clinical application of oseltamivir, a proportion of outpatients and inpatients developed the oseltamivir resistance (Hayden 2009; Nguyen et al., 2012; Gubareva et al., 2017). In particular, when people with waning immunity use long-term usage of antiviral treatment, it will result in an increased incidence of drug resistance (Krammer et al., 2018).

As an important alternative therapy for influenza treatment, traditional Chinese medicine (TCM) plays an essential role in global public health and is efficacious in reducing the patients fever and other related symptoms (Xiong et al., 2020). With the in-depth research, Chinese herbal medicines and related TCM extracts have been confirmed to exert their antiviral and immunoregulation, thereby effectively treating and preventing influenza (Xiong et al., 2020; Zhang et al., 2020). Furthermore, TCM can inhibit viral infection by directly targeting influenza viruses (Zhou et al., 2015). The active extracts and derivatives of the Chinese herbal medicines can inhibit oseltamivir-resistant influenza virus strains through broad-spectrum activity (Chung et al., 2014; Chung et al., 2015), and provide a novel treatment insight for the synergistic treatment of oseltamivir resistance with the Chinese medicine. When combined with oseltamivir, TCM does not affect the antiviral effect of oseltamivir, even increasing the drug absorption rate of oseltamivir (Zhang et al., 2019).

Traditional Chinese medicine injections (TCMIs), as the representative therapeutic interventions of antiviral drugs in the Chinese patent medicine, have been recognized and registered by the National Medical Products Administration and widely used in clinical practice for years in China. Although current clinical guidelines reported the clinical efficacy of oral Chinese patent medicines for treating influenza (Wu et al., 2020), there is still a lack of robust evidence regarding the TCMIs to date. Therefore, based on the actual clinical setting, we conduct this network meta-analysis to analyze the clinical efficacy and safety of various TCMIs combined with oseltamivir (TCMIs + oseltamivir) for influenza.

## 2 Methods

This systematic review and network meta-analysis were reported following the Preferred Reporting Items for Systematic Reviews and Meta-Analyses (PRISMA), PRISMA protocols, and the PRISMA-extension statement for network meta-analysis (Moher et al., 2009; Hutton et al., 2015; Shamseer et al., 2015). We registered and published our protocol for this network meta-analysis in the PROSPERO International Prospective Register of Systematic Reviews (https://www.crd.york.ac.uk/PROSPERO/) (registration number: CRD42021286994).

### 2.1 Eligibility Criteria

We included randomized controlled trials of patients with influenza and compared various TCMIs + oseltamivir and oseltamivir alone, without the language, geographical, and publication status restrictions. Eligible studies that focus on TCMIs were approved by the National Medical Products Administration with the detailed information on the drug, as of our retrieved date. Eligible treatment interventions were different TCMIs combined with oseltamivir, compared with oseltamivir alone as a control. There were no age, sex, or race restriction in our literature search.

Studies needed to be excluded when existing conditions were as follows: Patients suffering from serious life-threatening diseases and drug-resistant strains affect the research purposes for treating influenza. We also excluded ineligible studies, including retrospective cohort studies, population-based cross-sectional studies, case–control studies, and non-randomized trials. We excluded randomized controlled trials evaluating the non-oseltamivir antiviral treatment (amantadine, zanamivir, and ribavirin), oral traditional Chinese medicines (Chinese medicine decoction, granules, capsules, and pills), and non-pharmacologic treatments (acupuncture, moxibustion, massage, and electrical stimulation). Studies were excluded because of missing data, duplicate publications, or significant errors.

### 2.2 Search Strategy and Selection Criteria

The search included main Chinese databases [China National Knowledge Infrastructure, Wanfang Data Knowledge Service Platform, and VIP information resource integration service platform databases, and Chinese biomedical literature service system (SinoMed)] and English databases (PubMed, Embase, Cochrane Library, and Web of Science) without language restriction to find randomized controlled trials from database inception until October 2021. Clinicaltrials.gov, the Chinese clinical trial registry, and reference lists of included studies and relevant systematic reviews also were manually researched to cross-check the records, and to identify and supplement the potential eligible articles. The main searched terms (including MeSH words and text words) related to influenza, seasonal influenza, seasonal flu, flu, traditional Chinese medicine injection, and randomized controlled trials. Supplementary File S1 presents the detailed search strategy.

### 2.3 Data Extraction

Two investigators (YP and ZC) independently screened the titles and abstracts for the potentially eligible studies. We evaluated the full text of these articles in more detail by using the standardized and predesigned data extraction forms, according to our eligibility criteria, followed by a cross-check. When disagreements occurred in the literature screening and data extraction, the consensus was performed by discussion among two investigators or by consultation with a third reviewer (XL) if necessary. Investigators extracted information on the study characteristics (first author, publication time, study design, country, and duration), participant characteristics (age, sex, sample size, diseases, and disease course), intervention characteristics (interventions status, intervention duration, dose, drug class, and pharmaceutical company), and the description of outcomes (binary outcomes and dichotomous outcomes).

### 2.4 Outcome Assessment

We selected the outcomes that followed the importance to patients and crucial features of influenza, and the primary and secondary outcomes were informed after discussion by our professional panel. Selected primary outcomes included the response rate {calculated as [(number of total patients−number of invalid patients)/number of total patients] × 100%. Patients with unchanged or worsening symptoms (e.g., fever and cough) were considered invalid.}, disappearance time of fever, and disappearance time of cough. The negative time of nucleic acid and length of hospitalization could not be comprehensively assessed in this network comparison, due to the few numbers of included studies and missing information in certain TCMIs. Thus, secondary outcomes of this network meta-analysis included the negative time of nucleic acid and length of hospitalization as in this network meta-analysis, and safety with adverse events (dizziness, diarrhea, nausea, and vomiting).

### 2.5 Risk of Bias Assessment

Two investigators (XW and HC) independently assessed the quality of all eligible studies using the Cochrane Collaboration’s Risk of Bias tool (Higgins et al., 2011) to rate each item criterion of studies as either at low risk of bias, unclear risk of bias, and high risk of bias, across the following seven domains: random sequence generation, allocation concealment, blinding of participants and personnel, blinding of outcome assessment, incomplete outcome data, selective outcome reporting, and other bias. Any disagreements in the risk of bias assessment were resolved and evaluated by a discussion with a third investigator (XL).

### 2.6 Data Synthesis and Statistical Methods

We summarized the effect of interventions on dichotomous outcomes (response rate and safety: adverse events) using odds ratios (ORs) with corresponding 95% credible intervals (CrI) and continuous outcomes (i.e., improvement in fever and cough, negative time of nucleic acid, and length of hospitalization) using the mean differences (MDs) with corresponding 95% CrI. I^2^ statistics were used to assess the statistical heterogeneity in direct and indirect comparisons. We used a Bayesian random method based on the consistency assumption for network meta-analysis accounting for the combined direct within each study and indirect comparisons across studies. A vague prior was used for between-study heterogeneity, and MDs and log(OR) values were derived from the posterior distribution of the model. We choose the models that the Markov Chain Monte Carlo (MCMC) method with a weighted sample size to run in four Markov chains with set as least 2,00,000 iterations. All interventions were calculated by ranking probabilities using the surface under the curve cumulative ranking (SUCRA).

We performed sensitivity analyses that focus on children population to further explore and check the reliability and robustness of our findings. To resolve potential heterogeneity, we also conducted the univariate meta-regression (regressors: sample size, percentage male, mean age, and course of treatment) and used predictive mean matching imputations to fill the missing data for regressors. We performed the comparison-adjusted funnel plots by Egger’s and Begg’s tests to assess the publication bias in primary outcomes when 10 or more studies were included in the network comparison. The node splitting approach within each loop was not conducted to test the inconsistency and to obtain indirect estimates, as all including TCMI interventions were directly compared with oseltamivir alone. To assess the evidence certainty of interventions in all outcomes, we used the Grading of Recommendations, Assessment, Development, and Evaluation (GRADE) to identify the cumulative evidence, including the seven items (downgraded because of risk of bias, inconsistency, indirectness, imprecision, publication bias, intransitivity, and incoherence) and four grades of evidence certainty (very low, low, moderate, and high certainty of evidence) (Guyatt et al., 2011; Brignardello-Petersen et al., 2018).

We conducted this network meta-analysis with the Bayesian framework using R (version 4.0.5) for all outcomes, and the risk of bias was generated by RevMan (version 5.4) for all included studies.

## 3 Results

### 3.1 Search Results

**FIGURE 1:**
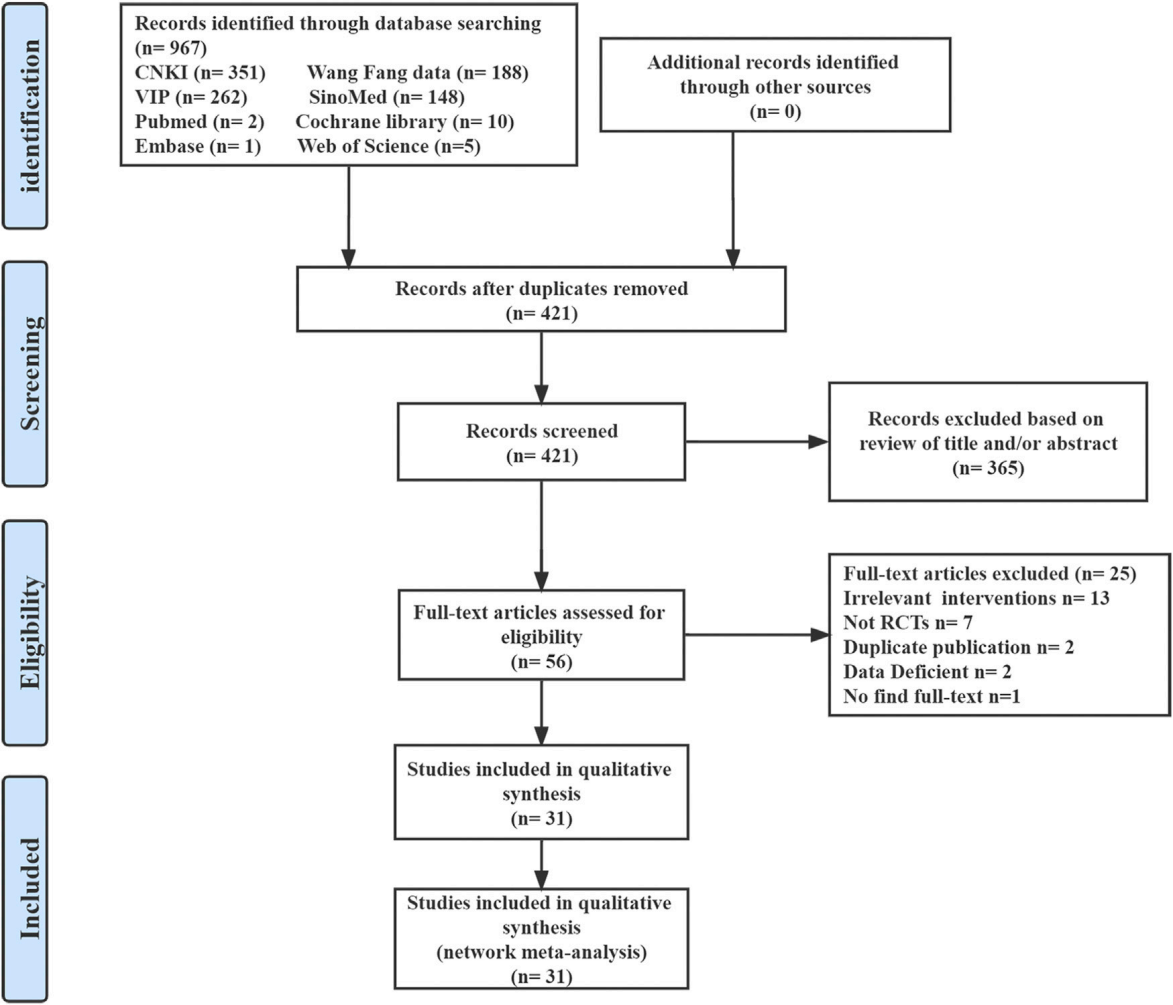
Summary of evidence search and selection.

We identified 967 initial articles from the English and Chinese database search and did not find any potentially additional studies from other sources. After title and abstract screening and full-text reviewing, 31 trials were deemed eligible for inclusion in our systematic review and network meta-analysis (Supplementary File S2). Figure 1 shows the detailed literature search process.

### 3.2 Study Characteristics

Supplementary Table S1 presents detailed characteristics of the included studies. Thirty-one eligible studies enrolled a total of 2,893 participants (TCMIs + oseltamivir: 1452; oseltamivir alone as control: 1441) with a median sample size (93 participants) ranging from 36 to 200 participants, mean age ranging from 2.92 to 46.6 years, the percentage of male ranging from 42.5 to 64.4%, and average time of interventions ranging from 3 to 10 days. Supplementary Tables S2 and S3 list the detailed chemical and raw material characterizations of all involved TCMIs. All publications of eligible randomized trials were conducted in China. We evaluated various TCMIs + oseltamivir compared with oseltamivir alone in the treatment of influenza with four direct comparisons: Xiyanping injections combined with oseltamivir (XYP + oseltamivir), Reduning injections combined with oseltamivir (RDN + oseltamivir), Tanreqing injections combined with oseltamivir (TRQ + oseltamivir), and Yanhuning injections combined with oseltamivir (YHN + oseltamivir).

### 3.3 Methodological Quality Assessment

Most of the included studies had the unclear risk of bias across all domains and proved at low risk of bias for at least one domain. Included studies reported the “random” assignment (32.26% present the specific methods of random sequence generation) and were judged to have the unclear risk of bias for blinding and allocation sequence concealment, and other bias in most or all studies. The risk of bias from the missing data posed the lowest risk (low risk of bias) due to all data being completely reported; 24 studies (77.42%) were at low risk of bias in the domains of selected outcomes reporting. The risk of bias assessments for each study are available in Supplementary Figures S1 and S2.

### 3.4 Outcomes

**FIGURE 2:**
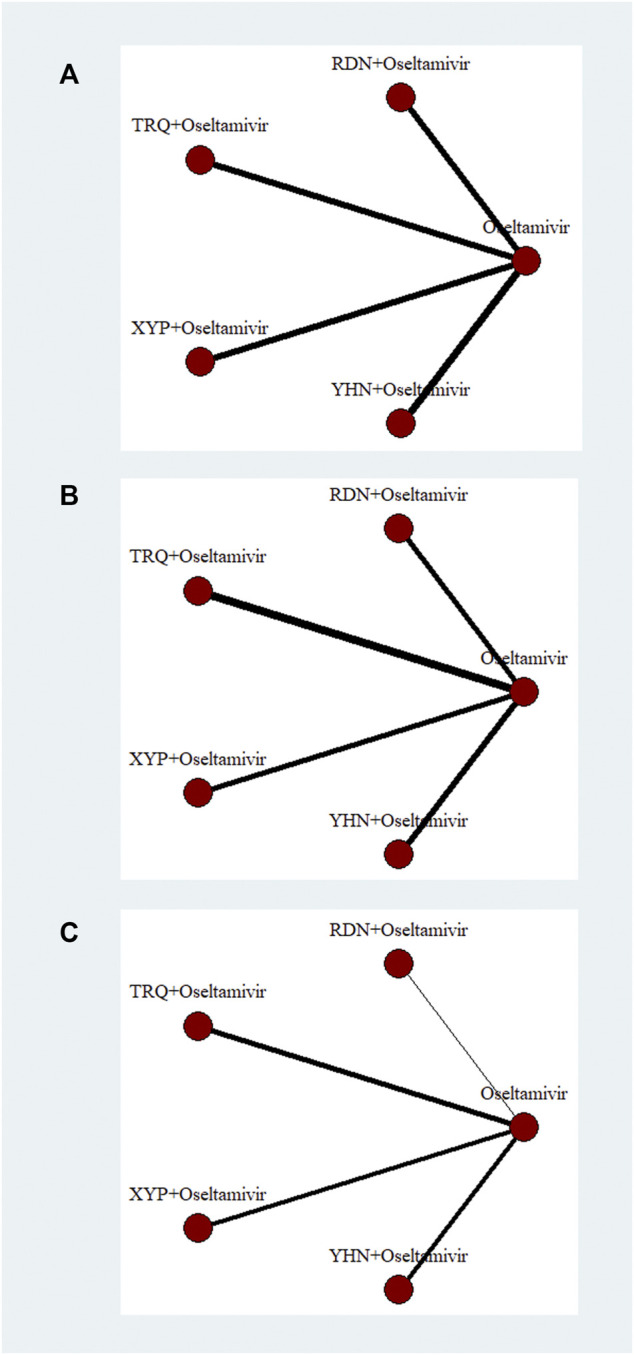
Network plots of primary outcomes. XYP, Xiyanping; RDN, Reduning; TRQ, Tanreqing; YHN, Yanhuning. **(A)** Response rate; **(B)** disappearance time of fever; **(C)** disappearance time of cough.

Figure 2 (primary outcomes) and Supplementary Figure S3 (other outcomes) show the network of interventions included in all outcome analyses for the direct comparisons between TCMIs + oseltamivir and oseltamivir.

### 3.5 Primary Outcomes

#### 3.5.1 Response Rate

**FIGURE 3:**
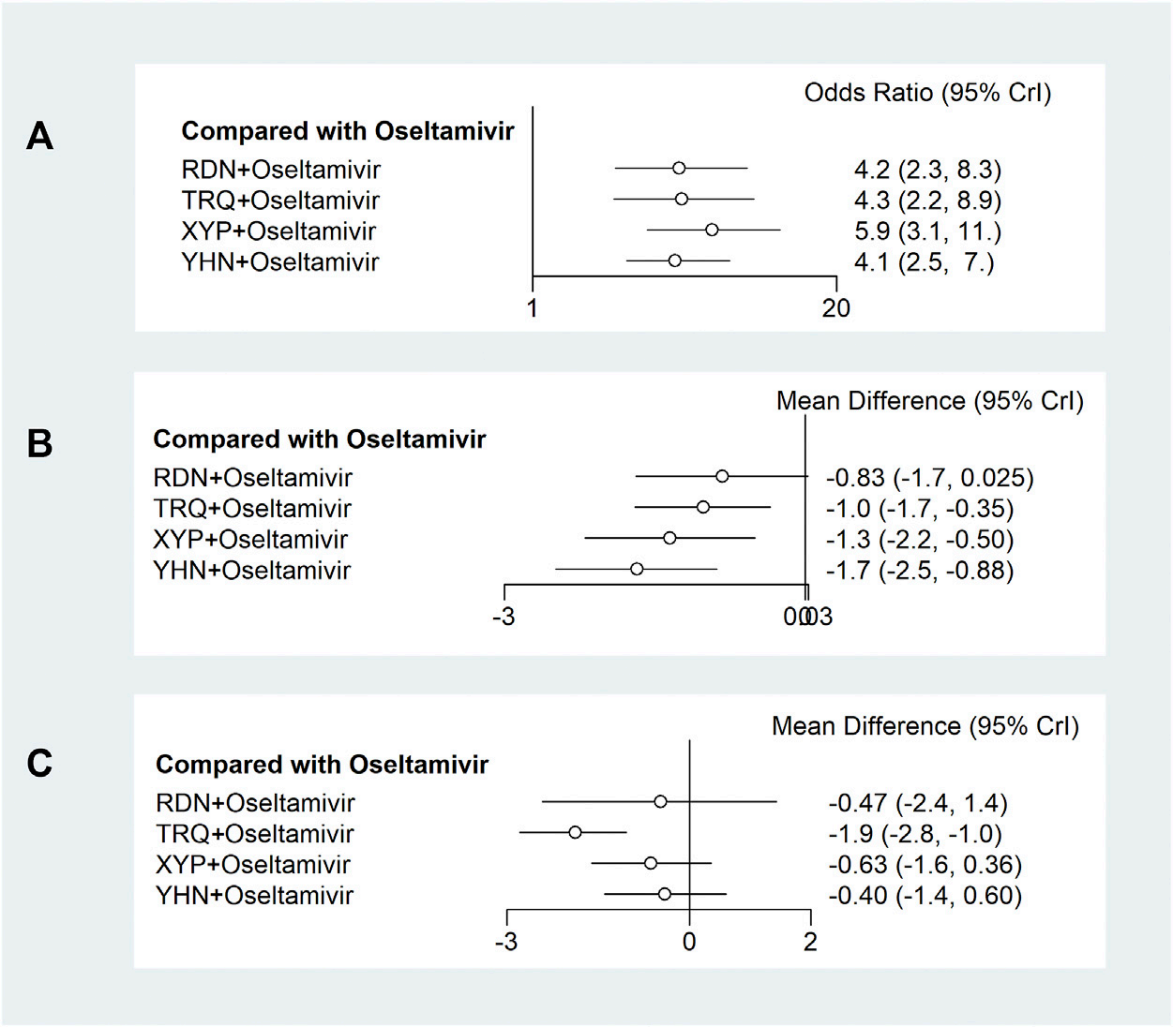
Forest plots of primary outcomes. XYP, Xiyanping; RDN, Reduning; TRQ, Tanreqing; YHN, Yanhuning. **(A)** Response rate; **(B)** disappearance time of fever; **(C)** disappearance time of cough. 95% CrI: 95% credible interval.

Twenty-five trials including 2,330 participants reported the response rate. Meta-analysis results with ranking according to the SUCRA value are as follows: XYP + oseltamivir (OR = 5.9, 95% CrI: 3.1 to 11; SUCRA = 0.82; very low certainty of evidence), TRQ + oseltamivir (OR = 4.3, 95% CrI: 2.2 to 8.9; SUCRA = 0.59; very low certainty of evidence), RDN + oseltamivir (OR = 4.2, 95% CrI: 2.3 to 8.3; SUCRA = 0.57; very low certainty of evidence), and YHN + oseltamivir (OR = 4.1, 95% CrI: 2.5 to 7; SUCRA = 0.52; very low certainty of evidence) were associated with significant difference in the response rate when compared with oseltamivir (Figure 3A, Supplementary Figure S4A, and Supplementary Table S4). Table 1 (top panel) shows that there were no significant differences among TCMIs + oseltamivir for the response rate.

**TABLE 1:** League table of response rate and disappearance time of fever.

	Response rate				
Disappearance time of fever	Oseltamivir	**4**.**24 (2**.**26**, **8**.**27)**	**4**.**35 (2.23**, **8**.**86)**	**5**.**87 (3**.**1**, **11**.**45)**	**4**.**07 (2**.**53**, **6**.**99)**
	−0.83 (−1.69, 0.03)	RDN + oseltamivir	1.03 (0.41, 2.66)	1.39 (0.55, 3.62)	0.95 (0.42, 2.23)
	−**1**.**02 (-1**.**7**, −**0**.**35)**	−0.19 (−1.28, 0.9)	TRQ + oseltamivir	1.34 (0.53, 3.51)	0.93 (0.4, 2.21)
	−**1**.**35 (**−**2**.**2**, −**0**.**5)**	−0.52 (−1.73, 0.7)	−0.33 (−1.4, 0.76)	XYP + oseltamivir	0.68 (0.31, 1.61)
	−**1**.**68 (**−**2**.**49**, −**0**.**88)**	−0.85 (−2.02, 0.31)	−0.66 (−1.71, 0.39)	−0.33 (−1.5, 0.85)	YHN + oseltamivir

Significant results are in bold.

Odds ratios with corresponding 95% credible intervals for response rate (upper triangle), and mean differences with corresponding 95% credible intervals for disappearance time of fever (lower triangle).

XYP, Xiyanping; RDN, Reduning; TRQ, Tanreqing; YHN, Yanhuning.

#### 3.5.2 Disappearance Time of Fever

Twenty-four trials including 2,194 participants reported the disappearance time of fever. Meta-analysis results with SUCRA showed that YHN + oseltamivir (MD = −1.7, 95% CrI: −2.5 to −0.88; SUCRA = 0.89; low certainty of evidence), XYP + oseltamivir (MD = −1.3, 95% CrI: −2.2 to −0.5; SUCRA = 0.71; low certainty of evidence), and TRQ + oseltamivir (MD = −1.0, 95% CrI: −1.7 to −0.35; SUCRA = 0.5; low certainty of evidence) were more efficacious than oseltamivir with significant difference (Figure 3B, Supplementary Figure S4B, and Supplementary Table S4). Table 1 (under panel) shows that there were no significant differences among TCMIs + oseltamivir in the disappearance time of fever.

#### 3.5.3 Disappearance Time of Cough

Fourteen trials including 1,290 participants reported the disappearance time of cough. Meta-analysis results with SUCRA showed that TRQ + oseltamivir (MD = −1.9, 95% CrI: −2.8 to −1; SUCRA = 0.97; low-certainty of the evidence) was more efficacious than oseltamivir with significant difference (Figure 3C, Supplementary Figure S4C, and Supplementary Table S4). Table 2 (top panel) shows that TRQ + oseltamivir reduced the duration of cough symptoms with a high probability than YHN + oseltamivir.

**TABLE 2:** League table of disappearance time of cough.

	Disappearance time of cough				
	Oseltamivir	−0.47 (−2.42, 1.43)	**−1**.**88 (−2**.**79**, **−1**.**04)**	−0.63 (−1.6, 0.36)	−0.4 (−1.4, 0.6)
		RDN + oseltamivir	−1.41 (−3.55, 0.67)	−0.16 (−2.3, 2.02)	0.07 (−2.12, 2.24)
			TRQ + oseltamivir	1.25 (−0.02, 2.6)	**1**.**48 (0**.**17**, **2**.**84)**
				XYP + oseltamivir	0.23 (−1.17, 1.62)
					YHN + oseltamivir

Significant results are in bold.

Mean differences with corresponding 95% credible intervals for disappearance time of cough (upper triangle).

XYP, Xiyanping; RDN, Reduning; TRQ, Tanreqing; YHN, Yanhuning.

#### 3.5.4 Biplot of Primary Outcomes

**FIGURE 4:**
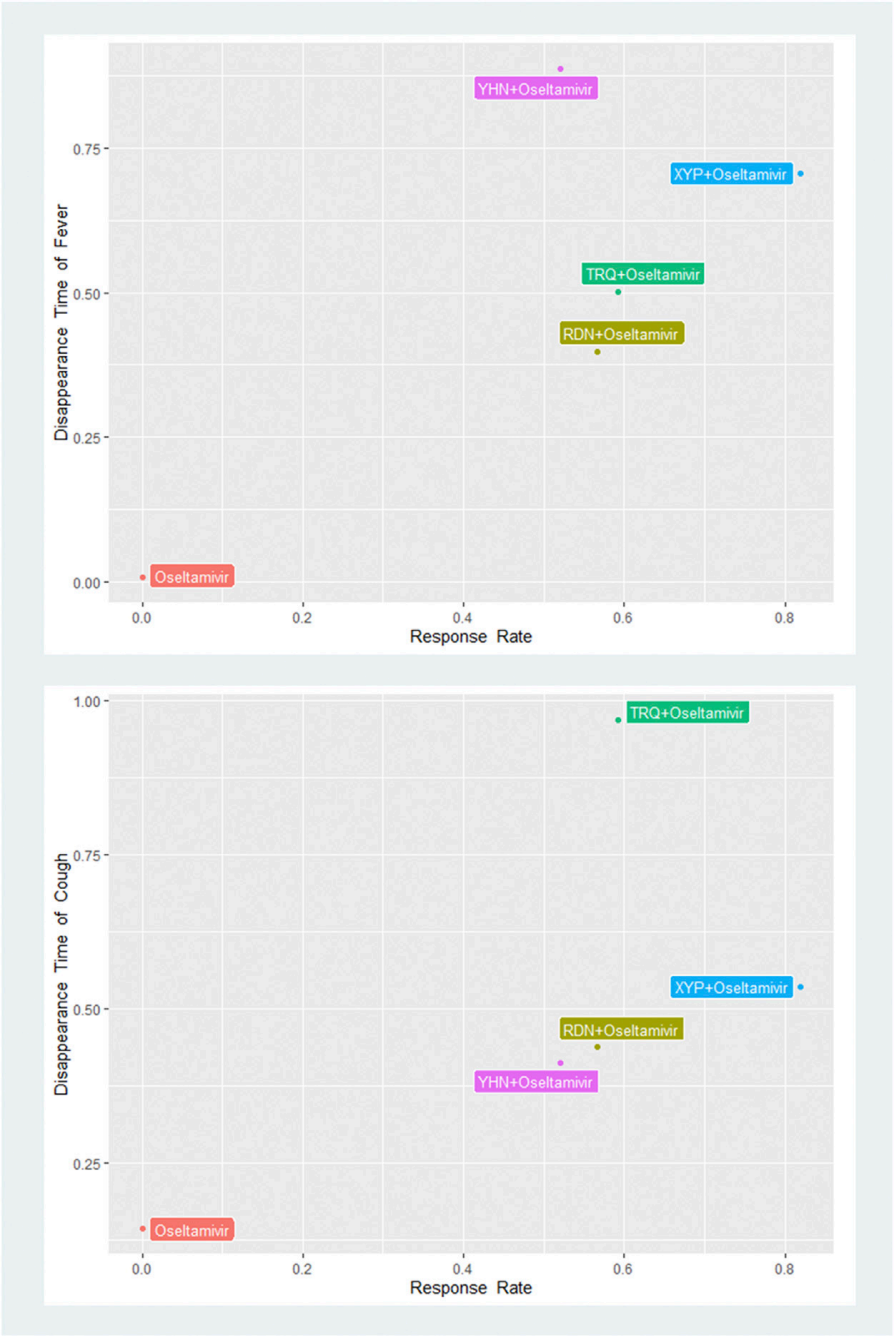
SUCRA biplots of primary outcomes. XYP, Xiyanping; RDN, Reduning; TRQ, Tanreqing; YHN, Yanhuning. SUCRA, the surface under the curve cumulative ranking.

Based on the SUCRA value of the network analysis results in primary outcomes, we performed the biplots that combined the response rate with the disappearance time of fever and cough to find the best interventions with combined effect for influenza. The results showed that XYP + oseltamivir (combination response rate and disappearance time of fever: 0.58) and TRQ + oseltamivir (combination response rate and disappearance time of cough: 0.57) had the highest combined SUCRA effect (Figure 4 and Supplementary Table S5).

### 3.6 Other Outcomes

#### 3.6.1 Length of Hospitalization

Nine trials including 678 participants reported the length of hospitalization. Three TCMIs + oseltamivir has significant differences compared with oseltamivir (Supplementary Figure S5A): RDN + oseltamivir (MD = −1.6, 95% CrI: −3.1 to −0.014), TRQ + oseltamivir (MD = −1.6, 95% CrI: −2.8 to −0.25), and XYP + oseltamivir (MD = −2.0, 95% CrI: −3.1 to −0.89). There were no significant differences between these TCMIs + oseltamivir (Supplementary Table S6).

#### 3.6.2 Negative Time of Nucleic Acid

Five trials including 557 participants reported a negative time of nucleic acid. YHN + oseltamivir (MD = −2.4, 95% CrI: −4.5 to −0.27) have significant differences compared with oseltamivir in Supplementary Figure S5B. There were no significant differences between involved TCMIs + oseltamivir (Supplementary Table S6).

#### 3.6.3 Safety

Ten trials including 878 participants reported adverse events. Meta-analysis showed TRQ + oseltamivir in total adverse events with the wide 95%Crl for the unstable estimates. Therefore, compared with oseltamivir, there were no significant differences among involved interventions for total adverse events, dizziness, diarrhea, nausea, and vomiting (Supplementary Figure S6). We summarized the detail information of safety in Supplementary Table S7 to supplement: XYP + oseltamivir (4 trials, 12 AEs), RDN + oseltamivir (1 trial, 3 AEs), TRQ + oseltamivir (1 trial, 2 AEs), YHN + oseltamivir (4 trial, 28 AEs), and oseltamivir (10 trial, 38 AEs). In all reported AEs, diarrhea was reported in XYP + oseltamivir and YHN + oseltamivir with higher incidence rate than in other interventions.

### 3.7 Additional Analyses

We performed the sensitivity analysis for populations based on children in this current study with sufficient data. In sensitivity analysis, the pooled results showed significant changes in the response rate (OR of TRQ + oseltamivir become 9.4) for including studies that focus on children and found no substantial change in other interventions (Supplementary Figure S7). Furthermore, we conducted the biplots of sensitivity analysis results to find the potential interventions with the highest combined SUCRA value: TRQ + oseltamivir with 0.71 in the combined response rate and disappearance time of cough, and YHN + oseltamivir with 0.55 in the combined response rate and disappearance time of fever (Supplementary Figure S8 and Supplementary Table S5).

Network meta-regression for primary outcomes showed that regressors had no statistically significant effects in most interventions. However, we found some heterogeneity in TRQ + oseltamivir (the disappearance time of fever: course of treatment; disappearance time of cough: course of treatment and age). Supplementary Table S8 summarizes the results of meta-regression for details.

### 3.8 Publication Bias

**FIGURE 5:**
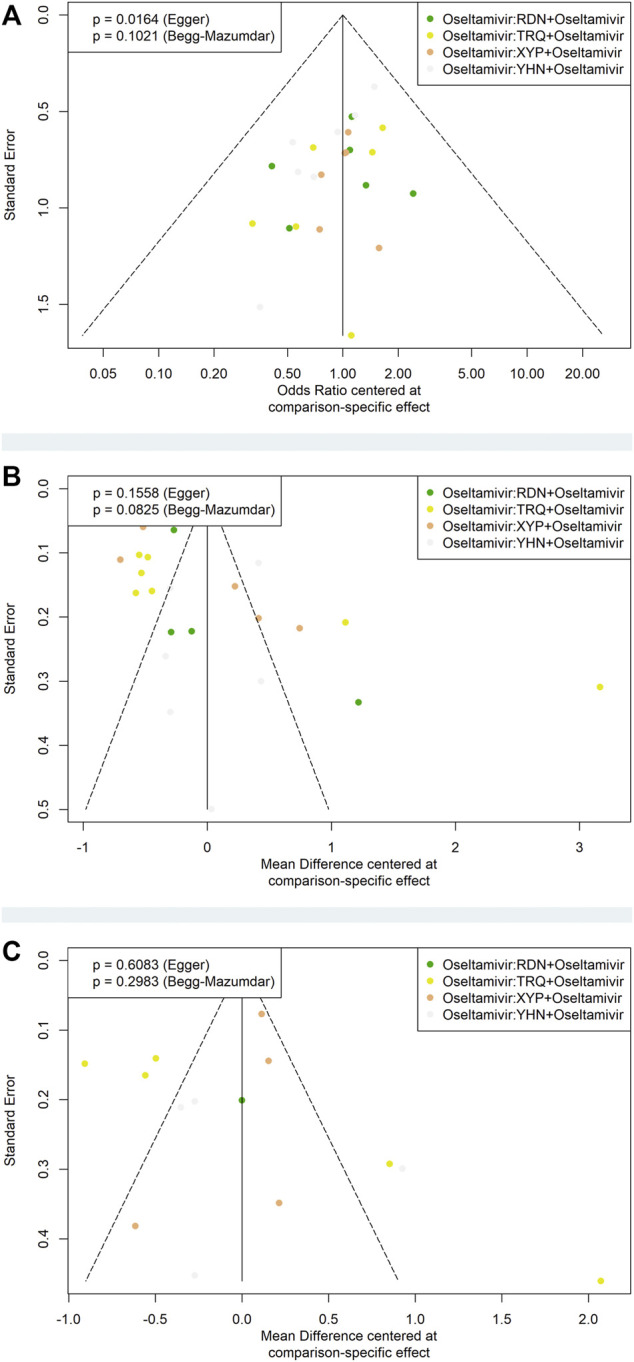
Funnel plots of primary outcomes. XYP, Xiyanping; RDN, Reduning; TRQ, Tanreqing; YHN, Yanhuning. **(A)** Response rate; **(B)** disappearance time of fever; **(C)** disappearance time of cough.

We conducted funnel plots using Begg’s and Egger’s tests to identify the potential publication bias in primary outcomes. The results of funnel plots showed that there were no significant asymmetry distributions in the disappearance time of fever and cough (Figure 5). We found some asymmetry distributions in the response rate (Egger: *p* = 0.0164).

## 4 Discussion

### 4.1 Principal Findings

Our systematic review and network meta-analysis, including 31 trials with 2,893 participants, provide a comprehensive overview of the evidence regarding the effectiveness and safety of various TCMIs + oseltamivir for influenza. In brief, low to very low certainty of evidence showed that XYP + oseltamivir, TRQ + oseltamivir, and YHN + oseltamivir probably appeared to be more effective than oseltamivir alone, after weighing the results of sensitivity analysis and meta-regression. Among them, results demonstrated potential therapeutic benefits of TRQ + oseltamivir for reducing cough, and XYP + oseltamivir and YHN + oseltamivir for improving fever in people with influenza, based on the interventions ranking with the SUCRA value and combined SUCRA value. We may have been unable to adequately assess the length of hospitalization and negative time of nucleic because of the evidence with insufficient included studies involving all interventions. Harms were still unclear, with no significant differences between TCMIs + oseltamivir over oseltamivir in the observed adverse events, while XYP + oseltamivir and YHN + oseltamivir may increase the risk of diarrhea due to a higher incidence rate than others. Taken together, these results in benefits and harms from our systematic review have some implications for TCM clinical practice in treating influenza.

### 4.2 Relation and Comparison With Other Studies

To our knowledge, this network meta-analysis compares the effectiveness and safety of various TCMIs + oseltamivir and oseltamivir alone for influenza first and in a comprehensive manner. Previously published systematic reviews of TCM for treating patients with influenza concluded that integrated traditional Chinese and western medicine probably had greater reduction in the time of fever and other symptoms over western medicine alone (Wang et al., 2014; Li et al., 2016). This in turn could lead to uncertainty and imprecision about outcomes because of clinical heterogeneity in interventions between different TCM treatments (e.g., decoction, self-prescription, soup, powder, and mixed TCM treatments) in these systematic reviews. Therefore, we focused on TCMIs combined with oseltamivir vs. oseltamivir alone across more RCTs to resolve the heterogeneity from interventions in our systematic reviews. It is noteworthy that in the clinical practice guideline on treating influenza with Chinese patent medicines (Wu et al., 2020), there was a lack of evidence for TCMIs with oseltamivir for influenza. TRQ, as one of the common TCMIs, could be beneficial for reducing the flu symptoms as observed by a previous meta-analysis published in 2019 (Qu et al., 2020). As evidence about other TCMIs remained insufficient, we evaluated various TCMIs more comprehensively in this systematic review. Our findings based on absolute estimates of important clinical outcomes of various TCMIs were important and timely for influenza, and provided support for the treatment regimen to TCM guidelines.

### 4.3 Policy Implications

For patients who have influenza symptoms, the combined administration of TCMIs with oseltamivir may have beneficial treatment effects in fever and cough disappearance time. XYP could effectively inhibit the function of influenza virus hemagglutinin to block viral replication, and induce immune regulation through AKT and NF-κB signaling pathways (Wang et al., 2019). Moreover, multicomponent anti-influenza bioactive components in TRQ inhibited influenza virus replication *in vivo* by inhibiting hemagglutinin and neuraminidase of influenza A virus to play a role in the anti-influenza effects (Zhu et al., 2018). Also, harms in rare and high risk cases may not be detected in this network meta-analysis. The potential risk of adverse events (e.g., diarrhea) that may be caused by TCMIs in our findings should not deter their clinical use for treating influenza. Our results can assist in clinical practice guidelines of TCM and update TCM treatment for influenza.

Clinical physicians must carefully weigh the potential benefits and harms in the TCMI treatment progress, and weigh against influenza patients’ comorbidities and willingness to use TCMIs. Discussing the therapeutic regimen with patients considering and receiving TCMIs is of great importance during treatment. Furthermore, future research necessitates large numbers of participants and high evidence in TCMIs to facilitate the detection of the potential harms; it was also important for physicians and patients.

### 4.4 Strengths and Limitations

Our systematic review focuses on updated TCM treatments for influenza and incorporates the best and latest evidence on various TCMIs combined with oseltamivir to facilitate and refine the current TCM guidelines. This network meta-analysis was conducted by a professional team of clinical physicians and methodological experts to ensure expertise in the overall review process. The strengths include a comprehensive search strategy with explicit eligibility criteria and adjudication of risk of bias by two investigators with uniform criteria. As all including TCMI interventions were compared with oseltamivir directly, estimates were not affected by transitivity and consistency. For potential clinical heterogeneity that may affect our results, to the best of our efforts, we conducted sensitivity analysis and multiple covariant meta-regression as additional analyses to diminish this concern. We also comprehensively evaluated the primary outcomes based on the combined SUCRA value to provide more strong evidence for our findings. Apart from TRQ + oseltamivir in the response rate, we did not find other interventions with significant changes among original analysis and sensitivity analysis, which present our results more robustness in most interventions. We also use funnel plots to evaluate the publication bias by two tests (Begg’s and Egger) in each comparison.

Despite these strengths, our study had some limitations. The main limitations of methodological quality assessment in most of the included studies were the unclear risk of bias in domains of blinding and allocation sequence concealment. Although without geographical restrictions in the retrieval process, all eligible included studies in this systematic review were conducted in mainland China, reducing the generalizability of our results. In meta-regression, confounders (age and intervention time) may remain in this network meta-analysis and affect the stability of TRQ + oseltamivir results based on the unavoidable clinical heterogeneity. Moreover, various disease severities of influenza and different flu viruses were the important heterogeneity for our results, but we do not identify the adequate data from the primary literature to explore. The measurement methods of the effectiveness may be different among different studies, which may affect our results with potential biases. We also found less precise estimation in OR with a wide 95% CrI for TRQ + oseltamivir in the sensitivity analysis. Some asymmetry distributions were found in the funnel plot of the response rate. Insufficient data on the length of hospitalization and negative time of nucleic acid were reported in several eligible trials for full analysis, which means results might not be as robust for the clinic and require additional clinical studies on these outcomes.

## 5 Conclusions

Evidence from this network meta-analysis showed that TCMIs + oseltamivir only partially improve symptoms in patients with influenza. Overall therapeutic efficacy and safety are inconclusive, based on low to very low certainty of evidence and no definitive evidence of safety, weighing the potential benefits and harms. Therefore, TCMIs should be used with great care in clinical practice and require further research for more high-quality evidence.

## Data Availability

The raw data supporting the conclusions of this article will be made available by the authors, without undue reservation.
