# Supplementary material for: Traditional Chinese Medicine Injections Combined With Oseltamivir for Influenza: Systematic Review and Network Meta-Analysis
Source: Front Pharmacol. 2022 Jul 22;13:848770. doi: 10.3389/fphar.2022.848770 (PMC9355026; doi:10.3389/fphar.2022.848770)
Supplement: Supplementary file 1 [file DataSheet1.docx]

**Supplementary Material**

**Contents of Supplementary Figures, Tables, Files, and PRISMA checklist**

[Supplementary Figure 1 Risk of Bias Summary 3](#_Toc100844011)

[Supplementary Figure 2 Risk of Bias of Included Studies 4](#_Toc100844012)

[Supplementary Figure 3 Network Plots of Other Outcomes 5](#_Toc100844013)

[Supplementary Figure 4 Ranking of Primary Outcomes 6](#_Toc100844014)

[Supplementary Figure 5 Forest Plots of Secondary Outcomes 7](#_Toc100844015)

[Supplementary Figure 6 Forest Plots of Safety 8](#_Toc100844016)

[Supplementary Figure 7 Forest Plots of Sensitivity Analysis 9](#_Toc100844017)

[Supplementary Figure 8 Biplots of Sensitivity Analysis 10](#_Toc100844018)

[Supplementary Table 1 Summary of All Included Studies 11](#_Toc100844019)

[Supplementary Table 2 Detailed Chemical Characterizations of Traditional Chinese Medicine Injections 17](#_Toc100844020)

[Supplementary Table 3 Raw material Characteristics of Included Studies 19](#_Toc100844021)

[Supplementary Table 4 Grading of recommendations, assessment, development, and evaluation in primary outcomes 25](#_Toc100844022)

[Supplementary Table 5 Combined SUCRA value of Biplots for Primary Outcomes 26](#_Toc100844023)

[Supplementary Table 6 League Table of secondary outcomes 26](#_Toc100844024)

[Supplementary Table 7 Detailed information for Safety 27](#_Toc100844025)

[Supplementary Table 8 Meta regression for Primary Outcomes 28](#_Toc100844026)

[Supplementary File 1 The Detailed Search Strategy 31](#_Toc100844027)

[Supplementary File 2 References of Included Studies 33](#_Toc100844028)

[Supplementary PRISMA Checklist 36](#_Toc100844029)

# Supplementary Figure 1 Risk of Bias Summary


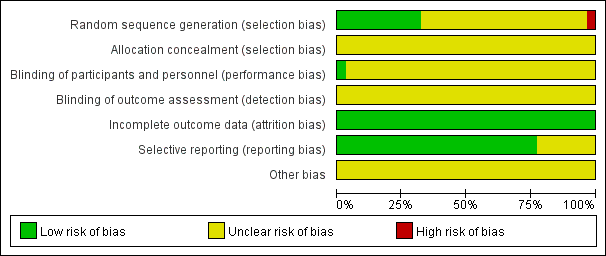


# Supplementary Figure 2 Risk of Bias of Included Studies


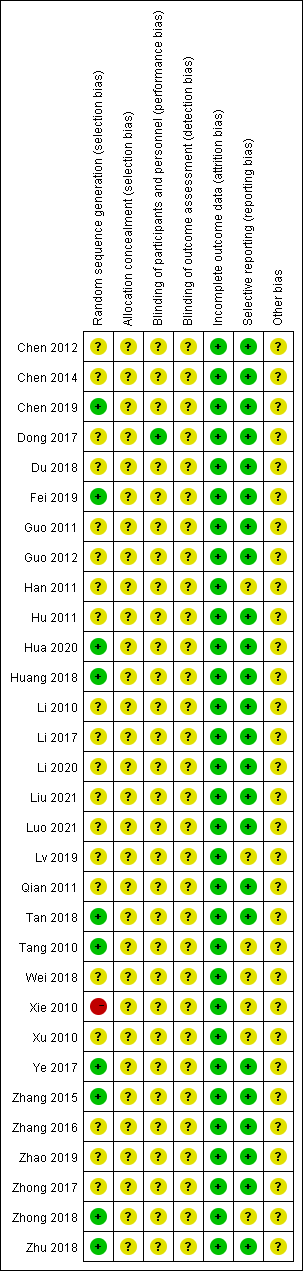


# Supplementary Figure 3 Network Plots of Other Outcomes


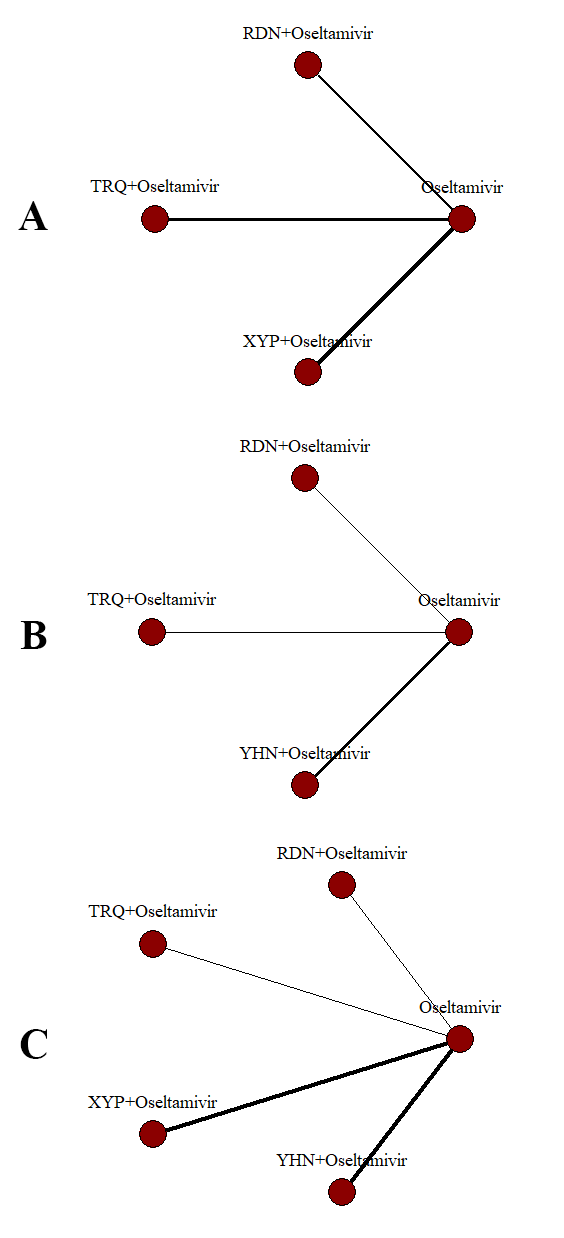


XYP: Xiyanping; RDN: Reduning; TRQ: Tanreqing; YHN: Yanhuning.

A: length of hospitalization; B: negative Time of nucleic acid; C: safety for total adverse events.

# Supplementary Figure 4 Ranking of Primary Outcomes


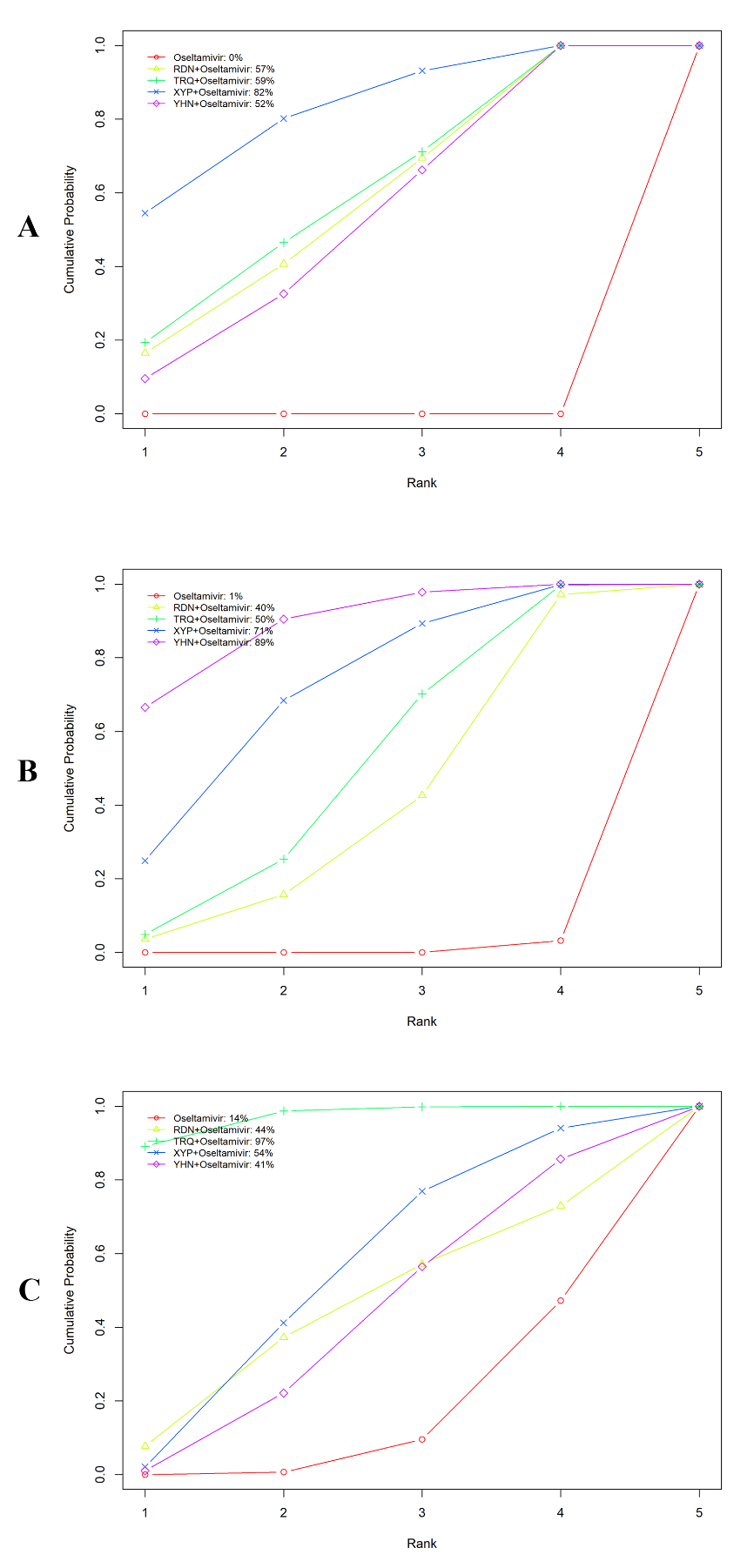


XYP: Xiyanping; RDN: Reduning; TRQ: Tanreqing; YHN: Yanhuning.

A: response rate; B: disappearance time of fever; C: disappearance time of cough.

# Supplementary Figure 5 Forest Plots of Secondary Outcomes


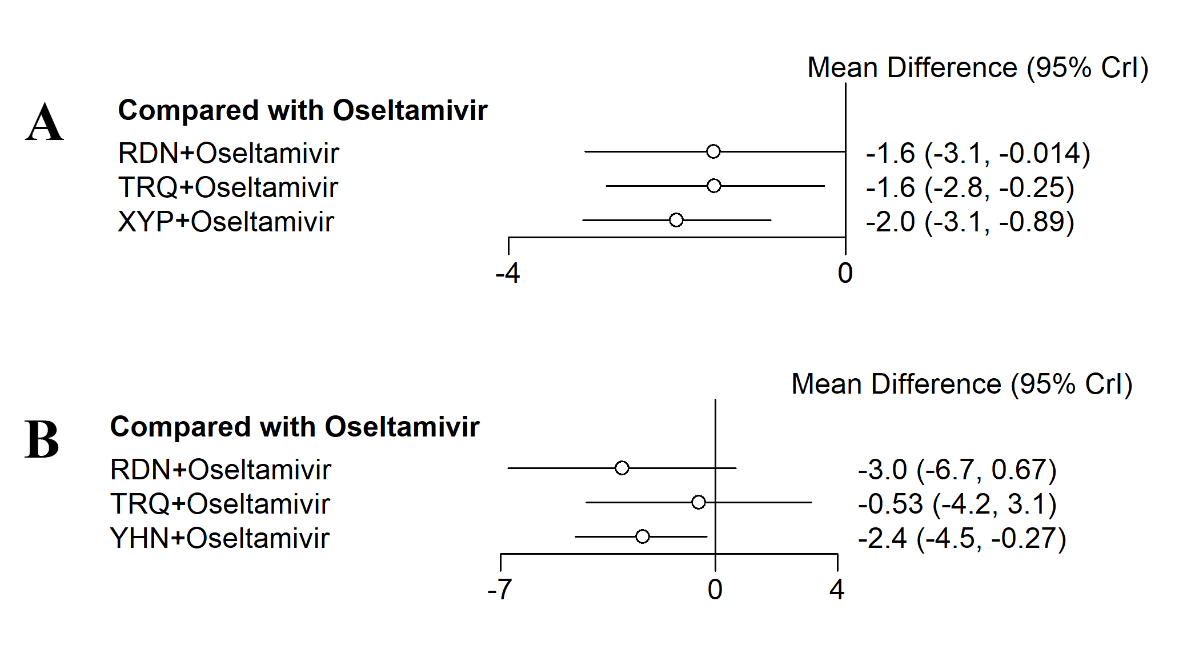


XYP: Xiyanping; RDN: Reduning; TRQ: Tanreqing; YHN: Yanhuning.

A: length of hospitalization; B: negative Time of nucleic acid

# Supplementary Figure 6 Forest Plots of Safety


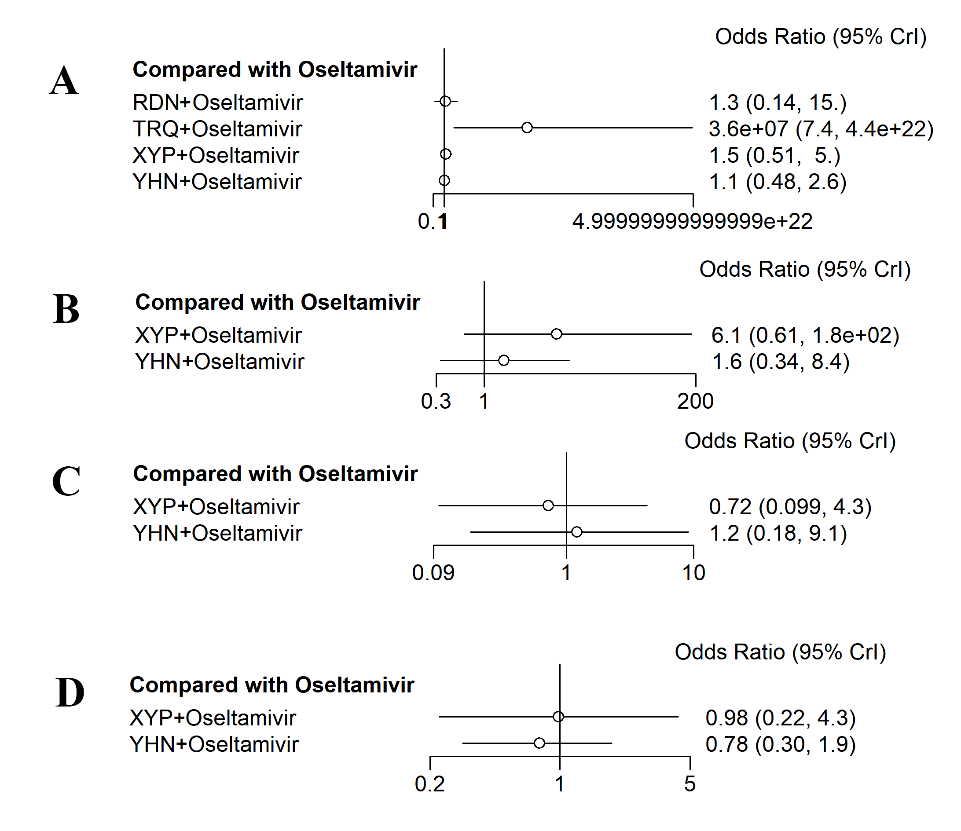


XYP: Xiyanping; RDN: Reduning; TRQ: Tanreqing; YHN: Yanhuning.

A: safety for total adverse events; B: dizziness; C: diarrhea; D: nausea and vomiting.

# Supplementary Figure 7 Forest Plots of Sensitivity Analysis


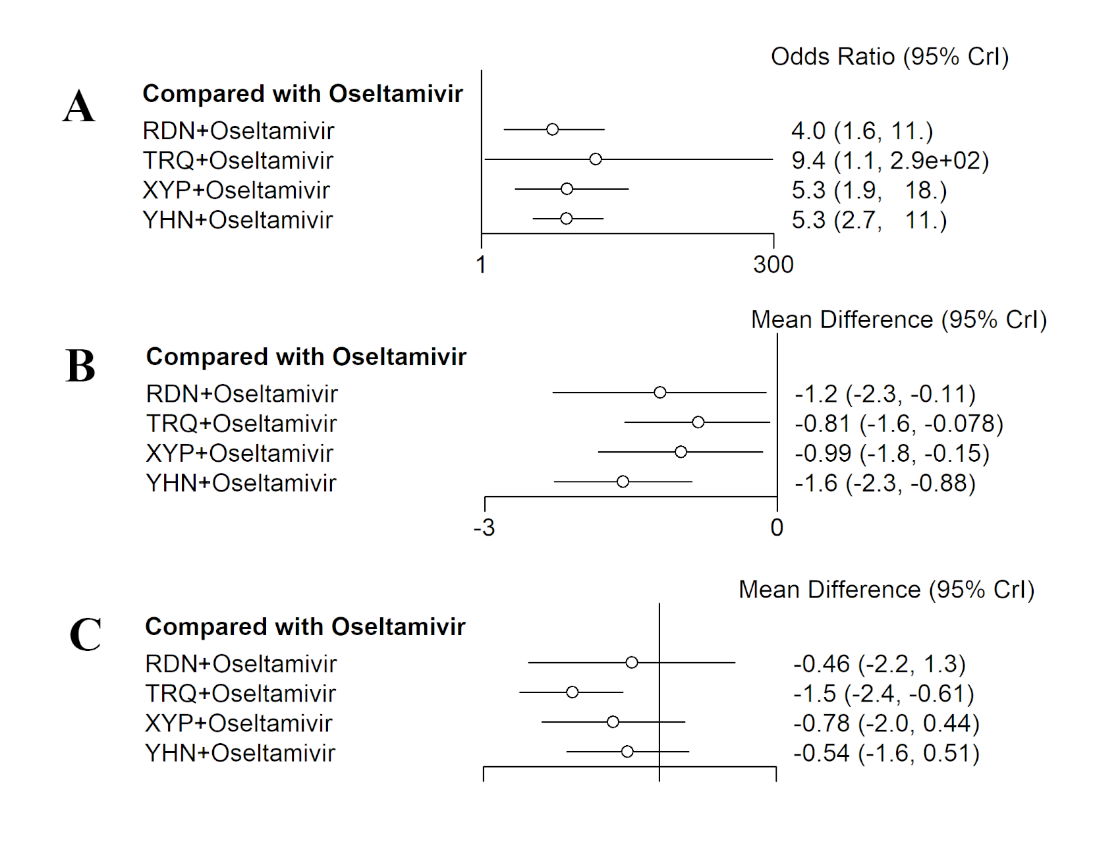


XYP: Xiyanping; RDN: Reduning; TRQ: Tanreqing; YHN: Yanhuning.

A: response rate; B: disappearance time of fever; C: disappearance time of cough.

# Supplementary Figure 8 Biplots of Sensitivity Analysis


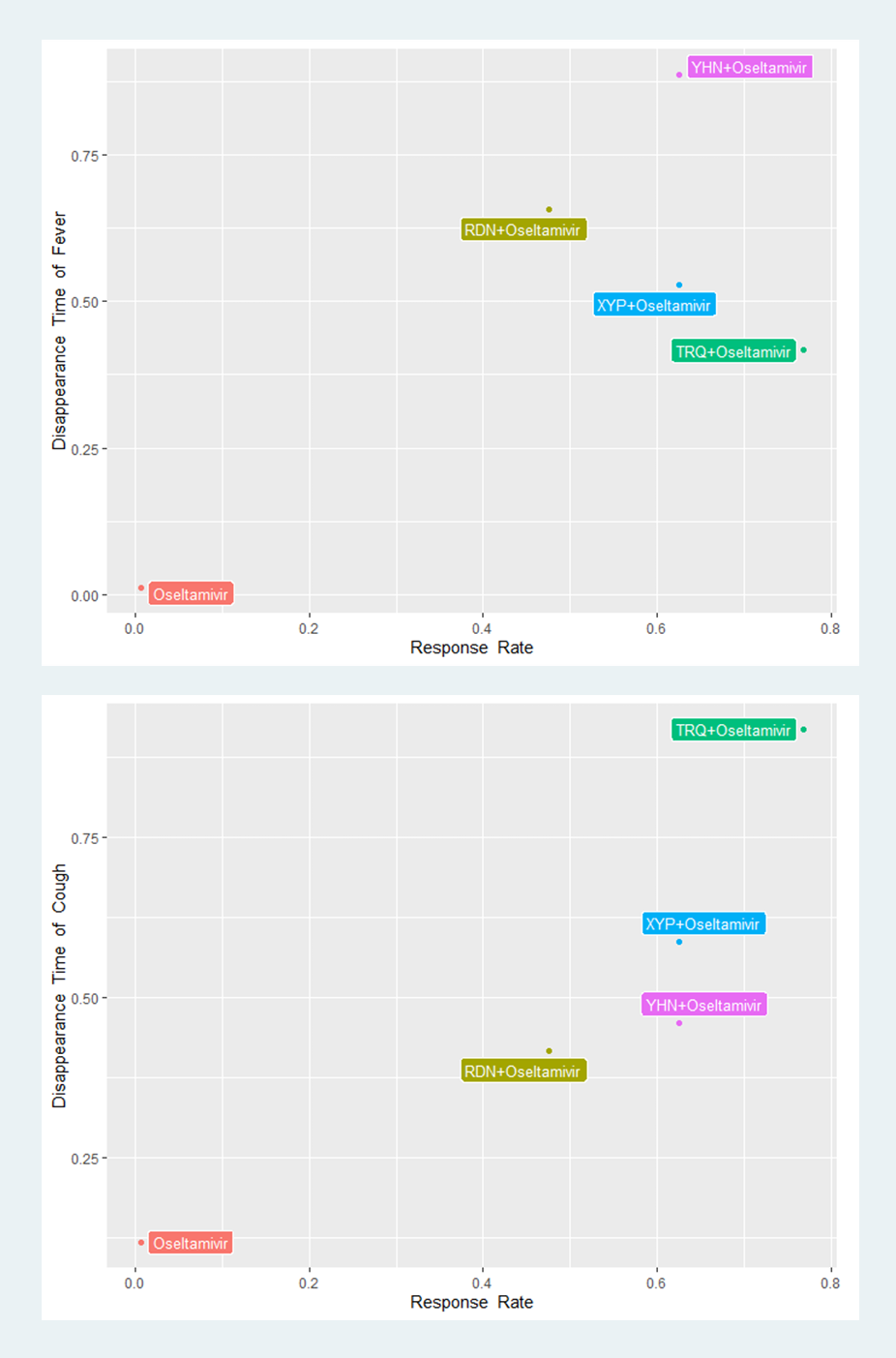


XYP: Xiyanping; RDN: Reduning; TRQ: Tanreqing; YHN: Yanhuning.

# Supplementary Table 1 Summary of All Included Studies

| **Author Year** | **Interventions** | | **Sample size** | | **Male** | | **Female** | | **Age (years)** | | **Detail information of dosage** | | **Course of treatment** | **Outcomes** | **Flu viruses** | **Influenza seasons** | **Hemispheres** |
| --- | --- | --- | --- | --- | --- | --- | --- | --- | --- | --- | --- | --- | --- | --- | --- | --- | --- |
|  | T | C | T | C | T | C | T | C | T | C | T | C |  |  |  |  |  |
| Guo 2012 | RDN+Oseltamivir | Oseltamivir | 43 | 43 | 20 | 22 | 23 | 21 | 29.2±15.0 | 30.0±15.1 | RDN (20ml/day) + Oseltamivir (150mg/day) | Oseltamivir (150mg/day) | 4 days | ②④ | Various flu viruses | All seasons | Northern hemisphere (China) |
| Hu 2011 | RDN+Oseltamivir | Oseltamivir | 40 | 38 | 18 | 20 | 22 | 18 | 3.98±2.39 | 4.12±3.42 | RDN (0.5ml/kg) + Oseltamivir | Oseltamivir | 7 days | ①②③④ | Influenza A (H1N1) | Winter | Northern hemisphere (China) |
| Hua 2020 | RDN+Oseltamivir | Oseltamivir | 65 | 55 | 30 | 35 | 25 | 30 | 5.9±2.3 | 5.9±2.6 | RDN (3-5 years: 0.3-0.8 ml/kg, not exceed 10 mL/day; 6-10 years: 10ml/day; 11-13 years: 15 ml/day; 14-17 years: 20ml/day) + Oseltamivir (6-12 months: 50mg/day; 1-13 years:  ≤15 kg, 60mg/day; 15-23 kg, 90mag/day; 23-40 kg, 120 mg/day;≥ 40 kg，150mg/day; 14+ years: 150 mg/day) | Oseltamivir (6-12 months: 50mg/day; 1-13 years:  ≤15 kg, 60mg/day; 15-23 kg, 90mag/day; 23-40 kg, 120 mg/day;≥ 40 kg，150mg/day; 14+ years: 150 mg/day) | 7 days | ①②⑤⑥ | Various flu viruses | All seasons | Northern hemisphere (China) |
| Lv 2019 | RDN+Oseltamivir | Oseltamivir | 29 | 29 | 14 | 12 | 15 | 17 | 2.97±1.89 | 2.86±1.82 | RDN (0.5ml/kg.d, not exceed 10ml/day) + Oseltamivir (＜15kg: 60mg/day; 15-23kg: 90mg/day; 23-40kg: 120mg/day.) | Oseltamivir (＜15kg: 60mg/day; 15-23kg: 90mg/day; 23-40kg: 120mg/day.) | 5 days | ① | Various flu viruses | All seasons | Northern hemisphere (China) |
| Xu 2010 | RDN+Oseltamivir | Oseltamivir | 82 | 80 | 46 | 45 | 36 | 35 | 18.3 (15-50) | 18.1 (15-48) | RDN (20ml/day) + Oseltamivir (150mg/day) | Oseltamivir (150mg/day) | 5 days | ① | Various flu viruses | Autumn | Northern hemisphere (China) |
| Ye 2017 | RDN+Oseltamivir | Oseltamivir | 50 | 50 | 28 | 26 | 22 | 24 | 27.02±7.09 | 28.14±7.11 | RDN (20ml/day) + Oseltamivir (150mg/day) | Oseltamivir (150mg/day) | 3 days | ①② | Various flu viruses | Spring | Northern hemisphere (China) |
| Zhao 2019 | RDN+Oseltamivir | Oseltamivir | 54 | 54 | 26 | 29 | 28 | 25 | 35.5±4.4 | 34±3.6 | RDN (20ml/day) + Oseltamivir (150mg/day) | Oseltamivir (150mg/day) | 3 days | ①② | Various flu viruses | All seasons | Northern hemisphere (China) |
| Chen 2012 | TRQ+Oseltamivir | Oseltamivir | 60 | 60 | 78 | | 42 | | 3-15 | 3-15 | TRQ (0.5ml/kg) + Oseltamivir | Oseltamivir | 5 days | ②③④ |  |  |  |
| Guo 2011 | TRQ+Oseltamivir | Oseltamivir | 69 | 69 | - | - | - | - | 15-46 | 15-46 | TRQ (20ml/day) + Oseltamivir (150mg/day) | Oseltamivir (150mg/day) | 5 days | ①② | Influenza A (H1N1) | Autumn and Winter | Northern hemisphere (China) |
| Han 2011 | TRQ+Oseltamivir | Oseltamivir | 22 | 21 | 22 | | 20 | | 35.5 (18-63) | 35.5 (18-63) | TRQ (40ml/day) + Oseltamivir (150mg/day) | Oseltamivir (150mg/day) | 5 days | ① |  |  |  |
| Li 2010 | TRQ+Oseltamivir | Oseltamivir | 55 | 55 | 28 | 32 | 27 | 23 | 31.35 (15-41) | 30.77 (17-40) | TRQ (20ml/day) + Oseltamivir | Oseltamivir | 7-14 days | ①②③ | Influenza A (H1N1) | Summer and Autumn | Northern hemisphere (China) |
| Luo 2021 | TRQ+Oseltamivir | Oseltamivir | 40 | 40 | 18 | 16 | 22 | 24 | 46.82±3.51 | 46.39±3.74 | TRQ (20ml/day) + Oseltamivir (150mg/day) | Oseltamivir (150mg/day) | 3 days | ①② | Various flu viruses | Autumn and Winter | Northern hemisphere (China) |
| Qian 2011 | TRQ+Oseltamivir | Oseltamivir | 25 | 29 | 11 | 16 | 14 | 13 | 40.91±19.81 | 41.22±15.62 | TRQ (20ml/day) + Oseltamivir (150mg/day) | Oseltamivir (150mg/day) | 10 days | ②④⑤ | Influenza A (H1N1) | Autumn and Winter | Northern hemisphere (China) |
| Xie 2010 | TRQ+Oseltamivir | Oseltamivir | 44 | 43 | 25 | 23 | 19 | 20 | 16 (13-18) | 16 (13-18) | TRQ (20～30mL/day)+ Oseltamivir (23-40kg, 120mg/day; ＞40kg,150mg/day) | Oseltamivir (23-40kg, 120mg/day; ＞40kg,150mg/day) | 5 days | ① | Influenza A (H1N1) | Autumn | Northern hemisphere (China) |
| Zhang 2015 | TRQ+Oseltamivir | Oseltamivir | 60 | 60 | - | - | - | - | - | - | TRQ (0.3～0.5mL/kg) + Oseltamivir (＜15kg, 60mg/day; 15-23kg, 90mg/day; 24-40kg, 120mg/day) | Oseltamivir (＜15kg, 60mg/day; 15-23kg, 90mg/day; 24-40kg, 120mg/day) | - | ②③⑥ | Various flu viruses | All seasons | Northern hemisphere (China) |
| Zhang 2016 | TRQ+Oseltamivir | Oseltamivir | 30 | 30 | 19 | 16 | 11 | 14 | 4.17±2.49 | 3.72±2.74 | TRQ (0.3～0.5mL/kg) + Oseltamivir (＜15kg, 60mg/day; 15-23kg, 90mg/day; 24-40kg, 120mg/day) | Oseltamivir (＜15kg, 60mg/day; 15-23kg, 90mg/day; 24-40kg, 120mg/day) | - | ②③ | Various flu viruses | Autumn, Winter and Spring | Northern hemisphere (China) |
| Zhong 2017 | TRQ+Oseltamivir | Oseltamivir | 59 | 59 | 37 | 39 | 22 | 20 | 3.2±0.8 | 3.5±0.6 | TRQ (0.5ml/kg) + Oseltamivir (＜15kg, 60mg/day; 15-23kg, 90mg/day; ≥24kg, 120mg/day) | Oseltamivir (＜15kg, 60mg/day; 15-23kg, 90mg/day; ≥24kg, 120mg/day) |  | ①②③④ | Various flu viruses | All seasons | Northern hemisphere (China) |
| Chen 2019 | XYP+Oseltamivir | Oseltamivir | 31 | 31 | 0 | 0 | 31 | 31 | 37.24±8.62 | 36.58±7.36 | XYP (5-10ml/day) + Oseltamivir (150mg/day) | Oseltamivir (150mg/day) | 5 days | ①②③④⑥ | Various flu viruses | All seasons | Northern hemisphere (China) |
| Du 2018 | XYP+Oseltamivir | Oseltamivir | 48 | 48 | - | - | - | - | - | - | XYP (0.2-0.4mg/kg) + Oseltamivir (4mg/kg) | Oseltamivir (4mg/kg) | 5 days | ①②③ | Various flu viruses | All seasons | Northern hemisphere (China) |
| Huang 2018 | XYP+Oseltamivir | Oseltamivir | 30 | 30 | 19 | 17 | 11 | 13 | 44.3±8.5 | 43.2±9 | XYP (10-20ml/day) + Oseltamivir (150mg/day) | Oseltamivir (150mg/day) | 5 days | ①②③④⑥ | Influenza A | All seasons | Northern hemisphere (China) |
| Tan 2018 | XYP+Oseltamivir | Oseltamivir | 18 | 18 | 9 | 10 | 9 | 8 | 6.13±1.35 | 6.36±1.48 | XYP (0.4ml/kg) + Oseltamivir (4mg/kg) | Oseltamivir (4mg/kg) | 5 days | ①②④⑥ | Influenza A | Summer, Autumn and Winter | Northern hemisphere (China) |
| Tang 2010 | XYP+Oseltamivir | Oseltamivir | 30 | 30 | 19 | 16 | 11 | 14 | 20.5 (14-56) | 21.2 (14-60) | XYP (100-200mg/day) + Oseltamivir | Oseltamivir | - | ① | Influenza A (H1N1) | Autumn | Northern hemisphere (China) |
| Zhu 2018 | XYP+Oseltamivir | Oseltamivir | 32 | 32 | 16 | 16 | 16 | 16 | 4.5 (1-10) | 4.7 (1-12) | XYP (0.2-0.4mg/kg) + Oseltamivir (4mg/kg) | Oseltamivir | - | ①②③④⑥ | Various flu viruses | All seasons | Northern hemisphere (China) |
| Chen 2014 | YHN+Oseltamivir | Oseltamivir | 58 | 60 | - | - | - | - | 4.57±3.32 | 4.57±3.32 | YHN (5-10mg/kg) + Oseltamivir (＜15kg, 60mg/day; 15-23kg, 90mg/day; 24-40kg, 120mg/day) | Oseltamivir (＜15kg, 60mg/day; 15-23kg, 90mg/day; 24-40kg, 120mg/day) | - | ①②③⑥ | Various flu viruses | All seasons | Northern hemisphere (China) |
| Dong 2017 | YHN+Oseltamivir | Oseltamivir | 31 | 30 | 18 | 16 | 13 | 14 | 11.42±1.47 | 11.16±1.64 | YHN (40mg/day)+ Oseltamivir (75mg/day) | Oseltamivir (75mg/day) | 5 days | ①②⑤ | Influenza A (H1N1) | Summer, Autumn and Winter | Northern hemisphere (China) |
| Fei 2019 | YHN+Oseltamivir | Oseltamivir | 30 | 30 | 16 | 15 | 14 | 15 | 3.15±1.28 | 3.28±1.31 | YHN (10 mg/kg) + Oseltamivir (150mg/day) | Oseltamivir (150mg/day) | 5 days | ①②③ | Influenza A (H1N1) | All seasons | Northern hemisphere (China) |
| Li 2020 | YHN+Oseltamivir | Oseltamivir | 61 | 61 | 36 | 33 | 25 | 28 | 9.83±1.65 | 9.68±1.46 | YHN (40mg/day) + Oseltamivir (75mg/day) | Oseltamivir (75mg/day) | 5 days | ①②⑤ | Influenza A (H1N1) | All seasons | Northern hemisphere (China) |
| Li 2017 | YHN+Oseltamivir | Oseltamivir | 61 | 61 | - | - | - | - | 4.35±2.81 | 4.35±2.81 | YHN (5-10 mg/kg) + Oseltamivir (＜15kg, 60mg/day; 15-23kg, 90mg/day; 23-40kg, 120mg/day) | Oseltamivir (＜15kg, 60mg/day; 15-23kg, 90mg/day; 24-40kg, 120mg/day) | - | ②③⑥ | Various flu viruses | All seasons | Northern hemisphere (China) |
| Liu 2021 | YHN+Oseltamivir | Oseltamivir | 100 | 100 | 57 | 55 | 43 | 45 | 38±2.33 | 39±2.43 | YHN (240 mg/day) + Oseltamivir (150mg/day) | Oseltamivir (150mg/day) | 3 days | ①②③⑤⑥ | Influenza A and B | Autumn and Winter | Northern hemisphere (China) |
| Wei 2018 | YHN+Oseltamivir | Oseltamivir | 57 | 57 | 31 | 30 | 26 | 27 | 3.15±0.72 | 3.21±0.86 | YHN (5-10 mg/kg) + Oseltamivir (＜15kg, 60mg/day; 15-23kg, 90mg/day; 24-40kg, 120mg/day;＞40kg, 150 mg/day) | Oseltamivir (＜15kg, 60mg/day; 15-23kg, 90mg/day; 24-40kg, 120mg/day;＞40kg, 150 mg/day) | 5 days | ① | Various flu viruses | All seasons | Northern hemisphere (China) |
| Zhong 2018 | YHN+Oseltamivir | Oseltamivir | 38 | 38 | 20 | 19 | 18 | 19 | 11.31±1.24 | 11.07±1.57 | YHN (40mg/day) + Oseltamivir (75mg/day) | Oseltamivir (75mg/day) | 5 days | ①⑥ | Various flu viruses | Spring, Autumn and Winter | Northern hemisphere (China) |

XYP: Xiyanping; RDN: Reduning; TRQ: Tanreqing; YHN: Yanhuning.

T: treatment group; C: control group.

Outcomes: ①: response rate (based on the fever, cough, and other indicators);②: disappearance time of fever;③: disappearance time of cough;④: length of hospitalization;⑤: negative Time of nucleic acid;⑥: safety.

# Supplementary Table 2 Detailed Chemical Characterizations of Traditional Chinese Medicine Injections

| **Traditional Chinese medicine Injections*** | **Main chemical characterization** | **Molecular Formula** | **Test range (mg/ml) tested by HPLC** |
| --- | --- | --- | --- |
| Reduning injection | Neochlorogenic acid（5-caffeoylquinic acid） | C16H18O9 | 2.4-2.83 |
|  | Chlorogenic acid | C16H18O9 | 6.06-6.92 |
|  | 4-dicaffeoylquinic acid | C16H18O9 | 2.74-3.41 |
|  | Isochlorogenic acid B | C25H24O12 | 0.4-0.54 |
|  | Isochlorogenic acid A | C25H24O12 | 0.22-0.33 |
|  | Isochlorogenic acid C (4,5-Dicaffeoylquinic acid) | C25H24O12 | 0.35-0.52 |
|  | Shanzhiside | C16H24O11 | 0.25-0.39 |
|  | Geniposidic acid | C16H22O10 | 0.52-0.9 |
|  | Genipin 1-gentiobioside | C23H34O15 | 4.24-6.18 |
|  | Geniposide | C17H24O10 | 9.98-10.89 |
|  | Secoxyloganin | C17H24O11 | 0.82-1.01 |
| Xiyanping injection | Andrographolide sulfate A | C20H27O11S2Na2 | 0.351-0.356 |
|  | Andrographolide sulfate B | C20H28O8SNa | 0.33-0.34 |
|  | Andrographolide sulfate C | C20H28O8SNa | 0.731-0.801 |
|  | 9-dehydro-17-hydro-andrographolide | C20H29O5 | 0.792-0.81 |
| Yanhuning injection | Dehydroandrographolide succinate | C28H36O10 | 0.02-0.3 |
| Tanreqing injection | Protocatechuic acid | C7H6O4 | 0.00389-0.004 |
|  | Chlorogenic acid | C16H18O9 | 0.04126-0.04244 |
|  | Caffeic acid | C9H8O4 | 0.0605-0.06259 |
|  | Baicalin | C21H18O11 | 5.3398-5.4644 |
|  | Ursodeoxycholic acid | C24H40O4 | 5.1364-5.3245 |
|  | Chenodeoxycholic acid | C24H40O4 | 0.9764-9975 |

***References:**

Feng, S. X., Zhao, D., Hao, R., Zhou, T. Q., Wang, M. M., Li, J. S. (2016). Simultaneous determination of six compounds in Tanreqing injection using HPLC-UV-ELSD. Chin J Pharm Anal. 36, 278-284. doi:10.16155/j.0254-1793.2016.02.14.

Wang, A. H. (2015). Determination of Content of Potassium Sodium Pehydroandrographolide Succinate for Injection by HPLC. Str Pharm J. 27, 48-50.

Wu, S., Wang, X., Wu, Y. N., Liu, Q. A., Wu, J. X., Bi, Y. A., et al. (2014). Determination of eleven major components and fingerprint chromatography for Reduning injection by UPLC. Chin J Chin Mat Med. 39, 4804-4810.

Zhan, H. Z., Chen, W. K., Xiao, X. W., Yang, X. L., Luo, Y. H. (2012). HPLC simultaneous determination of four effective ingredients in Xiyanping injection. Chin J Pharm Anal. 32, 140-143. doi:10.16155/j.0254-1793.2012.01.015.

# Supplementary Table 3 Raw material Characteristics of Included Studies

| **Study** | **Formulation** | **Source*** | **Raw material of herbal drugs** | **Quality control reported? (Y/N)** | **Chemical analysis reported? (Y/N)** |
| --- | --- | --- | --- | --- | --- |
| Guo 2012 | Reduning injection | Suwei Biopharmaceutical Co., Ltd. | Artemisia annua L. [Asteraceae;Artemisia annua stem and leaf];Lonicera japonica Thunb. [Caprifoliaceae;Lonicera japonica flower bud]; Gardenia jasminoides J.Ellis [Rubiaceae; Gardenia jasminoides fruit] | Y - Prepared according to People's Republic of China Pharmacopoeia | Y – HPLC |
| Hu 2011 | Reduning injection | Jiangsu Kanion Pharmaceutical Co., Ltd. (SFDA approval number: Z20050217) | Artemisia annua L. [Asteraceae;Artemisia annua stem and leaf];Lonicera japonica Thunb. [Caprifoliaceae;Lonicera japonica flower bud]; Gardenia jasminoides J.Ellis [Rubiaceae; Gardenia jasminoides fruit] | Y - Prepared according to People's Republic of China Pharmacopoeia | Y – HPLC |
| Hua 2020 | Reduning injection | Jiangsu Kanion Pharmaceutical Co., Ltd. (SFDA approval number: Z20050217) | Artemisia annua L. [Asteraceae;Artemisia annua stem and leaf];Lonicera japonica Thunb. [Caprifoliaceae;Lonicera japonica flower bud]; Gardenia jasminoides J.Ellis [Rubiaceae; Gardenia jasminoides fruit] | Y - Prepared according to People's Republic of China Pharmacopoeia | Y – HPLC |
| Lv 2019 | Reduning injection | Jiangsu Kanion Pharmaceutical Co., Ltd. (SFDA approval number: Z20050217) | Artemisia annua L. [Asteraceae;Artemisia annua stem and leaf];Lonicera japonica Thunb. [Caprifoliaceae;Lonicera japonica flower bud]; Gardenia jasminoides J.Ellis [Rubiaceae; Gardenia jasminoides fruit] | Y - Prepared according to People's Republic of China Pharmacopoeia | Y – HPLC |
| Xu 2010 | Reduning injection | Jiangsu Kanion Pharmaceutical Co., Ltd. (SFDA approval number: Z20050217) | Artemisia annua L. [Asteraceae;Artemisia annua stem and leaf];Lonicera japonica Thunb. [Caprifoliaceae;Lonicera japonica flower bud]; Gardenia jasminoides J.Ellis [Rubiaceae; Gardenia jasminoides fruit] | Y - Prepared according to People's Republic of China Pharmacopoeia | Y – HPLC |
| Ye 2017 | Reduning injection | Jiangsu Kanion Pharmaceutical Co., Ltd. (SFDA approval number: Z20050217) | Artemisia annua L. [Asteraceae;Artemisia annua stem and leaf];Lonicera japonica Thunb. [Caprifoliaceae;Lonicera japonica flower bud]; Gardenia jasminoides J.Ellis [Rubiaceae; Gardenia jasminoides fruit] | Y - Prepared according to People's Republic of China Pharmacopoeia | Y – HPLC |
| Zhao 2019 | Reduning injection | Jiangsu Kanion Pharmaceutical Co., Ltd. (SFDA approval number: Z20050217) | Artemisia annua L. [Asteraceae;Artemisia annua stem and leaf];Lonicera japonica Thunb. [Caprifoliaceae;Lonicera japonica flower bud]; Gardenia jasminoides J.Ellis [Rubiaceae; Gardenia jasminoides fruit] | Y - Prepared according to People's Republic of China Pharmacopoeia | Y – HPLC |
| Chen 2012 | Tanreqing injection | Shanghai Kaibao Pharmaceutical Co., Ltd. (SFDA approval number: Z20030054) | Scutellaria baicalensis Georgi [Lamiaceae; Scutellaria baicalensis Georgi root]; Lonicera japonica Thunb. [Caprifoliaceae; Lonicera japonica flower bud]; Forsythia suspensa (Thunb.) Vahl [Oleaceae; Forsythia suspensa fruit]; Selenarctos thibetanus [G. Cuvier; bear bile substitutes]; Saiga tatarica Linnaeus; [Saiga tatarica Linnaeus substitutes] | Y - Prepared according to People's Republic of China Pharmacopoeia | Y – HPLC |
| Guo 2011 | Tanreqing injection | Shanghai Kaibao Pharmaceutical Co., Ltd. (SFDA approval number: Z20030054) | Scutellaria baicalensis Georgi [Lamiaceae; Scutellaria baicalensis Georgi root]; Lonicera japonica Thunb. [Caprifoliaceae; Lonicera japonica flower bud]; Forsythia suspensa (Thunb.) Vahl [Oleaceae; Forsythia suspensa fruit]; Selenarctos thibetanus [G. Cuvier; bear bile substitutes]; Saiga tatarica Linnaeus; [Saiga tatarica Linnaeus substitutes] | Y - Prepared according to People's Republic of China Pharmacopoeia | Y – HPLC |
| Han 2011 | Tanreqing injection | Shanghai Kaibao Pharmaceutical Co., Ltd. (SFDA approval number: Z20030054) | Scutellaria baicalensis Georgi [Lamiaceae; Scutellaria baicalensis Georgi root]; Lonicera japonica Thunb. [Caprifoliaceae; Lonicera japonica flower bud]; Forsythia suspensa (Thunb.) Vahl [Oleaceae; Forsythia suspensa fruit]; Selenarctos thibetanus [G. Cuvier; bear bile substitutes]; Saiga tatarica Linnaeus; [Saiga tatarica Linnaeus substitutes] | Y - Prepared according to People's Republic of China Pharmacopoeia | Y – HPLC |
| Li 2010 | Tanreqing injection | Shanghai Kaibao Pharmaceutical Co., Ltd. (SFDA approval number: Z20030054) | Scutellaria baicalensis Georgi [Lamiaceae; Scutellaria baicalensis Georgi root]; Lonicera japonica Thunb. [Caprifoliaceae; Lonicera japonica flower bud]; Forsythia suspensa (Thunb.) Vahl [Oleaceae; Forsythia suspensa fruit]; Selenarctos thibetanus [G. Cuvier; bear bile substitutes]; Saiga tatarica Linnaeus; [Saiga tatarica Linnaeus substitutes] | Y - Prepared according to People's Republic of China Pharmacopoeia | Y – HPLC |
| Luo 2021 | Tanreqing injection | Shanghai Kaibao Pharmaceutical Co., Ltd. (SFDA approval number: Z20030054) | Scutellaria baicalensis Georgi [Lamiaceae; Scutellaria baicalensis Georgi root]; Lonicera japonica Thunb. [Caprifoliaceae; Lonicera japonica flower bud]; Forsythia suspensa (Thunb.) Vahl [Oleaceae; Forsythia suspensa fruit]; Selenarctos thibetanus [G. Cuvier; bear bile substitutes]; Saiga tatarica Linnaeus; [Saiga tatarica Linnaeus substitutes] | Y - Prepared according to People's Republic of China Pharmacopoeia | Y – HPLC |
| Qian 2011 | Tanreqing injection | Shanghai Kaibao Pharmaceutical Co., Ltd. (SFDA approval number: Z20030054) | Scutellaria baicalensis Georgi [Lamiaceae; Scutellaria baicalensis Georgi root]; Lonicera japonica Thunb. [Caprifoliaceae; Lonicera japonica flower bud]; Forsythia suspensa (Thunb.) Vahl [Oleaceae; Forsythia suspensa fruit]; Selenarctos thibetanus [G. Cuvier; bear bile substitutes]; Saiga tatarica Linnaeus; [Saiga tatarica Linnaeus substitutes] | Y - Prepared according to People's Republic of China Pharmacopoeia | Y – HPLC |
| Xie 2010 | Tanreqing injection | Shanghai Kaibao Pharmaceutical Co., Ltd. (SFDA approval number: Z20030054) | Scutellaria baicalensis Georgi [Lamiaceae; Scutellaria baicalensis Georgi root]; Lonicera japonica Thunb. [Caprifoliaceae; Lonicera japonica flower bud]; Forsythia suspensa (Thunb.) Vahl [Oleaceae; Forsythia suspensa fruit]; Selenarctos thibetanus [G. Cuvier; bear bile substitutes]; Saiga tatarica Linnaeus; [Saiga tatarica Linnaeus substitutes] | Y - Prepared according to People's Republic of China Pharmacopoeia | Y – HPLC |
| Zhang 2015 | Tanreqing injection | Shanghai Kaibao Pharmaceutical CO.,LTD (SFDA approval number: Z20130025/Z20030054) | Scutellaria baicalensis Georgi [Lamiaceae; Scutellaria baicalensis Georgi root]; Lonicera japonica Thunb. [Caprifoliaceae; Lonicera japonica flower bud]; Forsythia suspensa (Thunb.) Vahl [Oleaceae; Forsythia suspensa fruit]; Selenarctos thibetanus [G. Cuvier; bear bile substitutes]; Saiga tatarica Linnaeus; [Saiga tatarica Linnaeus substitutes] | Y - Prepared according to People's Republic of China Pharmacopoeia | Y – HPLC |
| Zhang 2016 | Tanreqing injection | Shanghai Kaibao Pharmaceutical CO.,LTD (SFDA approval number: Z20130025/Z20030054) | Scutellaria baicalensis Georgi [Lamiaceae; Scutellaria baicalensis Georgi root]; Lonicera japonica Thunb. [Caprifoliaceae; Lonicera japonica flower bud]; Forsythia suspensa (Thunb.) Vahl [Oleaceae; Forsythia suspensa fruit]; Selenarctos thibetanus [G. Cuvier; bear bile substitutes]; Saiga tatarica Linnaeus; [Saiga tatarica Linnaeus substitutes] | Y - Prepared according to People's Republic of China Pharmacopoeia | Y – HPLC |
| Zhong 2017 | Tanreqing injection | Shanghai Kaibao Pharmaceutical CO.,LTD (SFDA approval number: Z20130025/Z20030054) | Scutellaria baicalensis Georgi [Lamiaceae; Scutellaria baicalensis Georgi root]; Lonicera japonica Thunb. [Caprifoliaceae; Lonicera japonica flower bud]; Forsythia suspensa (Thunb.) Vahl [Oleaceae; Forsythia suspensa fruit]; Selenarctos thibetanus [G. Cuvier; bear bile substitutes]; Saiga tatarica Linnaeus; [Saiga tatarica Linnaeus substitutes] | Y - Prepared according to People's Republic of China Pharmacopoeia | Y – HPLC |
| Chen 2019 | Xiyanping injection | Jiangxi Qingfeng Pharmaceutical Industry Co., Ltd. (SFDA approval number: Z20026249 | Andrographis paniculata (Burm.f.) Nees [Acanthaceae;Andrographis paniculata stem, leaf, flower] | Y - Prepared according to People's Republic of China Pharmacopoeia | Y – HPLC |
| Du 2018 | Xiyanping injection | Jiangxi Qingfeng Pharmaceutical Industry Co., Ltd. (SFDA approval number: Z20026249 | Andrographis paniculata (Burm.f.) Nees [Acanthaceae;Andrographis paniculata stem, leaf, flower] | Y - Prepared according to People's Republic of China Pharmacopoeia | Y – HPLC |
| Huang 2018 | Xiyanping injection | Jiangxi Qingfeng Pharmaceutical Industry Co., Ltd. (SFDA approval number: Z20026249 | Andrographis paniculata (Burm.f.) Nees [Acanthaceae;Andrographis paniculata stem, leaf, flower] | Y - Prepared according to People's Republic of China Pharmacopoeia | Y – HPLC |
| Tan 2018 | Xiyanping injection | Jiangxi Qingfeng Pharmaceutical Industry Co., Ltd. (SFDA approval number: Z20026249 | Andrographis paniculata (Burm.f.) Nees [Acanthaceae;Andrographis paniculata stem, leaf, flower] | Y - Prepared according to People's Republic of China Pharmacopoeia | Y – HPLC |
| Tang 2010 | Xiyanping injection | Jiangxi Qingfeng Pharmaceutical Industry Co., Ltd. (SFDA approval number: Z20026249 | Andrographis paniculata (Burm.f.) Nees [Acanthaceae;Andrographis paniculata stem, leaf, flower] | Y - Prepared according to People's Republic of China Pharmacopoeia | Y – HPLC |
| Zhu 2018 | Xiyanping injection | Jiangxi Qingfeng Pharmaceutical Industry Co., Ltd. (SFDA approval number: Z20026249 | Andrographis paniculata (Burm.f.) Nees [Acanthaceae;Andrographis paniculata stem, leaf, flower] | Y - Prepared according to People's Republic of China Pharmacopoeia | Y – HPLC |
| Chen 2014 | Yanhuning injection | Harbin Pharmaceutical Group Sanjing Jiabin Pharmaceutical Co., Ltd. (SFDA approval number: H20045421) | Andrographis paniculata (Burm.f.) Nees [Acanthaceae;Andrographis paniculata stem, leaf, flower] | Y - Prepared according to People's Republic of China Pharmacopoeia | Y – HPLC |
| Dong 2017 | Yanhuning injection | Sichuan Ziren Pharmaceutical Co., Ltd. (SFDA approval number: H20057890 | Andrographis paniculata (Burm.f.) Nees [Acanthaceae;Andrographis paniculata stem, leaf, flower] | Y - Prepared according to People's Republic of China Pharmacopoeia | Y – HPLC |
| Fei 2019 | Yanhuning injection | Yaopharma Co., Ltd. (SFDA approval number: H50021641 | Andrographis paniculata (Burm.f.) Nees [Acanthaceae;Andrographis paniculata stem, leaf, flower] | Y - Prepared according to People's Republic of China Pharmacopoeia | Y – HPLC |
| Li 2020 | Yanhuning injection | Guangdong Hongyuan Group Pharmaceutical Co., Ltd. (SFDA approval number: H20046406 | Andrographis paniculata (Burm.f.) Nees [Acanthaceae;Andrographis paniculata stem, leaf, flower] | Y - Prepared according to People's Republic of China Pharmacopoeia | Y – HPLC |
| Li 2017 | Yanhuning injection | NR | Andrographis paniculata (Burm.f.) Nees [Acanthaceae;Andrographis paniculata stem, leaf, flower] | Y - Prepared according to People's Republic of China Pharmacopoeia | Y – HPLC |
| Liu 2021 | Yanhuning injection | Yaopharma Co., Ltd. (SFDA approval number: H50021628 | Andrographis paniculata (Burm.f.) Nees [Acanthaceae;Andrographis paniculata stem, leaf, flower] | Y - Prepared according to People's Republic of China Pharmacopoeia | Y – HPLC |
| Wei 2018 | Yanhuning injection | NR | Andrographis paniculata (Burm.f.) Nees [Acanthaceae;Andrographis paniculata stem, leaf, flower] | Y - Prepared according to People's Republic of China Pharmacopoeia | Y – HPLC |
| Zhong 2018 | Yanhuning injection | Harbin Medisan Pharmaceutical Co., Ltd. (SFDA approval number: H20061057 | Andrographis paniculata (Burm.f.) Nees [Acanthaceae;Andrographis paniculata stem, leaf, flower] | Y - Prepared according to People's Republic of China Pharmacopoeia | Y – HPLC |

*SFDA: State Food and Drug Administration; NR: not reported.

# Supplementary Table 4 Grading of recommendations, assessment, development, and evaluation in primary outcomes

| **Primary Outcomes** | **Interventions** | **Effect (95%CrI)** | **Quality of evidence*** |
| --- | --- | --- | --- |
| **Response Rate** | XYP+Oseltamivir | 5.87 (3.1, 11.45) | Very low ^1,5^ |
|  | TRQ+Oseltamivir | 4.35 (2.23, 8.86) | Very low ^1,5^ |
|  | RDN+Oseltamivir | 4.24 (2.26, 8.27) | Very low ^1,5^ |
|  | YHN+Oseltamivir | 4.07 (2.53, 6.99) | Very low ^1,5^ |
| **Disappearance Time of Fever** | XYP+Oseltamivir | -1.35 (-2.2, -0.5) | Low^1^ |
|  | TRQ+Oseltamivir | -1.02 (-1.7, -0.35) | Low^1^ |
|  | RDN+Oseltamivir | -0.83 (-1.69, 0.03) | Very low^1,4^ |
|  | YHN+Oseltamivir | -1.68 (-2.49, -0.88) | Low^1^ |
| **Disappearance Time of Cough** | XYP+Oseltamivir | -0.63 (-1.6, 0.36) | Low^1^ |
|  | TRQ+Oseltamivir | -1.88 (-2.79, -1.04) | Low^1^ |
|  | RDN+Oseltamivir | -0.47 (-2.42, 1.43) | Very low^1,4^ |
|  | YHN+Oseltamivir | -0.4 (-1.4, 0.6) | Low^1^ |

*Reasons for downgrading direct evidence, indirect and Mixed estimates: 1. Downgraded because of Risk of Bias; 2. Downgraded because of Inconsistency; 3. Downgraded because of Indirectness; 4. Downgraded because of Imprecision; 5. Downgraded because of Publication Bias; 6. Downgraded because of Intransitivity; 7. Downgraded because of Incoherence. 95% CrI: 95% Credible Interval.

XYP: Xiyanping; RDN: Reduning; TRQ: Tanreqing; YHN: Yanhuning.

# Supplementary Table 5 Combined SUCRA value of Biplots for Primary Outcomes

| **Interventions** | **Disappearance Time of Fever combined with Response Rate** | | **Disappearance Time of Cough combined with Response Rate** | |
| --- | --- | --- | --- | --- |
|  | **Original network analysis results** | **Sensitivity analysis results** | **Original network analysis results** | **Sensitivity analysis results** |
| **Oseltamivir** | 0 | 0.000072762 | 0 | 0.000726738 |
| **RDN+Oseltamivir** | 0.225438517 | 0.311662893 | 0.248069877 | 0.197983634 |
| **TRQ+Oseltamivir** | 0.296751374 | 0.320061245 | **0.574569999** | **0.705508571** |
| **XYP+Oseltamivir** | **0.578820263** | 0.330364982 | 0.439086088 | 0.366538156 |
| **YHN+Oseltamivir** | 0.46220515 | **0.554831621** | 0.215371605 | 0.287889164 |

XYP: Xiyanping; RDN: Reduning; TRQ: Tanreqing; YHN: Yanhuning. Best combined SUCRA value are in bold.

# Supplementary Table 6 League Table of secondary outcomes

|  | **Length of Hospitalization** | | | | |
| --- | --- | --- | --- | --- | --- |
| **Negative Time of Nucleic Acid** | Oseltamivir | **-1.57**  **(-3.09, -0.01)** | **-1.56**  **(-2.84, -0.25)** | **-2.01**  **(-3.12, -0.89)** |  |
|  | -3.03  (-6.74, 0.67) | RDN+Oseltamivir | 0  (-2, 2.01) | -0.44  (-2.36, 1.46) |  |
|  | -0.53  (-4.19, 3.15) | 2.48  (-2.64, 7.69) | TRQ+Oseltamivir | -0.45  (-2.17, 1.24) |  |
|  |  |  |  | XYP+Oseltamivir |  |
|  | **-2.36**  **(-4.55, -0.27)** | 0.68  (-3.66, 4.96) | -1.83  (-6.15, 2.37) |  | YHN+oseltamivir |

XYP: Xiyanping; RDN: Reduning; TRQ: Tanreqing; yhn: yanhuning. mean differences with corresponding 95% credible intervals for length of hospitalization and negative time of nucleic acid. Significant results are in bold.

# Supplementary Table 7 Detailed information for Safety

| **Study Year** | **Interventions** |  | **Total adverse events** | **Simple siza** | **Dizziness** | **Diarrhea** | **Nausea and vomiting** | **Rash** | **Measles and itching** | **Chills** |
| --- | --- | --- | --- | --- | --- | --- | --- | --- | --- | --- |
| **Hua 2020** | RDN+Oseltamivir | | 3 | 65 |  |  |  |  |  |  |
| **Zhang 2015** | TRQ+Oseltamivir | | 2 | 60 |  |  |  | 1 |  | 1 |
| **Chen 2019** | XYP+Oseltamivir | | 4 | 31 | 0 | 2 | 2 |  |  |  |
| **Huang 2018** | XYP+Oseltamivir | | 4 | 30 | 2 | 1 | 1 |  |  |  |
| **Tan 2018** | XYP+Oseltamivir | | 3 | 18 | 1 | 1 | 1 |  |  |  |
| **Zhu 2018** | XYP+Oseltamivir | | 1 | 32 |  |  |  |  | 1 |  |
| **Chen 2014** | YHN+Oseltamivir | | 6 | 58 | 1 | 2 | 3 |  |  |  |
| **Li 2017** | YHN+Oseltamivir | | 6 | 12 |  |  |  |  |  |  |
| **Liu 2021** | YHN+Oseltamivir | | 12 | 100 | 1 | 5 | 6 |  |  |  |
| **Zhong 2018** | YHN+Oseltamivir | | 4 | 38 | 1 | 1 | 2 |  |  |  |
| **Hua 2020** | Oseltamivir | | 2 | 55 |  |  |  |  |  |  |
| **Zhang 2015** | Oseltamivir | | 0 | 60 |  |  |  |  |  |  |
| **Chen 2019** | Oseltamivir | | 2 | 31 | 1 |  | 1 |  |  |  |
| **Huang 2018** | Oseltamivir | | 5 | 30 | 2 | 1 | 2 |  |  |  |
| **Tan 2018** | Oseltamivir | | 2 | 18 | 1 |  | 1 |  |  |  |
| **Zhu 2018** | Oseltamivir | | 0 | 32 |  |  |  |  |  |  |
| **Chen 2014** | Oseltamivir | | 5 | 58 |  | 2 | 3 |  |  |  |
| **Li 2017** | Oseltamivir | | 5 | 12 |  |  |  |  |  |  |
| **Liu 2021** | Oseltamivir | | 14 | 100 | 3 | 2 | 9 |  |  |  |
| **Zhong 2018** | Oseltamivir | | 3 | 38 |  | 1 | 2 |  |  |  |

XYP: Xiyanping; RDN: Reduning; TRQ: Tanreqing; YHN: Yanhuning.

# Supplementary Table 8 Meta regression for Primary Outcomes

| **Response Rate** | | | | | | |
| --- | --- | --- | --- | --- | --- | --- |
|  | Interventions | 2.50% | 25% | 50% | 75% | 97.5% |
| **Mean age** | RDN+Oseltamivir | -0.95804 | -0.02879 | 0.44705 | 0.9312 | 1.9245 |
|  | TRQ+Oseltamivir | -1.5188 | -0.52306 | -0.0213 | 0.4795 | 1.4709 |
|  | XYP+Oseltamivir | -1.2813 | -0.43922 | -0.01349 | 0.4161 | 1.26 |
|  | YHN+Oseltamivir | -1.58522 | -0.89697 | -0.55944 | -0.2227 | 0.4522 |
| **Male** | RDN+Oseltamivir | -2.195 | -1.09355 | -0.53997 | -0.00427 | 1.0337 |
|  | TRQ+Oseltamivir | -2.8247 | -1.36574 | -0.65644 | 0.02668 | 1.3572 |
|  | XYP+Oseltamivir | -2.774 | -0.89182 | -0.01958 | 0.83658 | 2.623 |
|  | YHN+Oseltamivir | -1.2086 | -0.71599 | -0.46924 | -0.22766 | 0.2449 |
| **Simple size** | RDN+Oseltamivir | -2.313015 | -1.09475 | -0.49568 | 0.08948 | 1.2352 |
|  | TRQ+Oseltamivir | -3.656561 | -1.9645 | -1.16663 | -0.41997 | 0.9529 |
|  | XYP+Oseltamivir | -2.481561 | -0.84411 | -0.04153 | 0.77384 | 2.4843 |
|  | YHN+Oseltamivir | -1.390616 | -0.84322 | -0.57776 | -0.31669 | 0.1887 |
| **Course of disease** | RDN+Oseltamivir | -1.28425 | -0.57752 | -0.2207 | 0.1306 | 0.8162 |
|  | TRQ+Oseltamivir | -1.23203 | -0.73363 | -0.4793 | -0.2275 | 0.2666 |
|  | XYP+Oseltamivir | -7.56834 | -1.36317 | 0.4118 | 2.5586 | 13.107 |
|  | YHN+Oseltamivir | -0.63475 | 0.3447 | 0.8392 | 1.3389 | 2.3682 |
| **Disappearance Time of Fever** | | | | | | |
|  | Interventions | 2.50% | 25% | 50% | 75% | 97.5% |
| **Mean age** | RDN+Oseltamivir | -1.4353 | 0.008153 | 0.7466 | 1.4343 | 2.746 |
|  | TRQ+Oseltamivir | -7.8073 | -0.36829 | 0.0776 | 0.49672 | 3.2254 |
|  | XYP+Oseltamivir | -2.6362 | -1.42753 | -0.8879 | -0.28308 | 1.3529 |
|  | YHN+Oseltamivir | -2.713 | -1.30663 | 0.6018 | 0.08607 | 1.5642 |
| **Male** | RDN+Oseltamivir | -4.7713 | -2.3708 | -1.3634 | -0.35368 | 1.57909 |
|  | TRQ+Oseltamivir | -2.6219 | -1.6227 | -1.141 | -0.69312 | 0.34892 |
|  | XYP+Oseltamivir | -2.6855 | -0.5949 | 0.274 | 1.12898 | 2.86789 |
|  | YHN+Oseltamivir | -1.4259 | -0.6962 | -0.3335 | 0.02193 | 0.86096 |
| **Simple size** | RDN+Oseltamivir | -5.4481 | -2.8649 | -1.5304 | -0.3509 | 1.9484 |
|  | TRQ+Oseltamivir | -2.5339 | -1.3323 | -0.8058 | -0.2726 | 0.8092 |
|  | XYP+Oseltamivir | -1.4148 | 0.1177 | 0.9782 | 1.9153 | 3.546 |
|  | YHN+Oseltamivir | -1.4229 | -0.6663 | -0.2711 | 0.1306 | 1.0302 |
| **Course of disease** | RDN+Oseltamivir | -2.2243 | -1.0833 | -0.4709 | 0.1705 | 7.7739 |
|  | TRQ+Oseltamivir | -2.4944 | -1.4325 | -1.0881 | -0.7835 | -0.10849 |
|  | XYP+Oseltamivir | -13.0759 | -1.9509 | 1.1576 | 5.25 | 20.21777 |
|  | YHN+Oseltamivir | -2.2485 | -0.187 | 0.8484 | 1.8555 | 4.67026 |
| **Disappearance Time of Cough** | | | | | | |
|  | Interventions | 2.50% | 25% | 50% | 75% | 97.5% |
| **Mean age** | RDN+Oseltamivir | -16.3333 | -2.635207 | 0.4934 | 4.5405 | 34.1855 |
|  | TRQ+Oseltamivir | -4.7288 | -3.432439 | -2.7849 | -2.0803 | -0.5553 |
|  | XYP+Oseltamivir | -1.0181 | -0.083743 | 0.3147 | 0.7086 | 1.6131 |
|  | YHN+Oseltamivir | -1.0387 | -0.007388 | 0.4424 | 0.8892 | 1.9207 |
| **Male** | RDN+Oseltamivir | -67.9217 | -7.17794 | -0.6944 | 3.01425 | 16.4015 |
|  | TRQ+Oseltamivir | -3.1046 | -1.55983 | -0.835 | -0.08477 | 1.5824 |
|  | XYP+Oseltamivir | -5.2936 | -2.03587 | -0.4844 | 0.96931 | 3.9592 |
|  | YHN+Oseltamivir | -1.0865 | -0.03365 | 0.4154 | 0.85975 | 1.8441 |
| **Simple size** | RDN+Oseltamivir | -15.8566 | -2.8332 | 0.3377 | 4.1142 | 45.646 |
|  | TRQ+Oseltamivir | -3.4849 | -1.5024 | -0.5951 | 0.2919 | 2.3274 |
|  | XYP+Oseltamivir | -5.131 | -2.0749 | -0.6123 | 0.7006 | 3.6021 |
|  | YHN+Oseltamivir | -0.9301 | 0.1232 | 0.616 | 1.136 | 2.3595 |
| **Course of disease** | RDN+Oseltamivir | -38.0944 | -3.9541 | 0.2381 | 4.8099 | 32.0896 |
|  | TRQ+Oseltamivir | -2.4983 | -1.7399 | -1.3785 | -1.0129 | -0.2058 |
|  | XYP+Oseltamivir | -13.3984 | -3.3459 | -0.5398 | 2.2567 | 10.7546 |
|  | YHN+Oseltamivir | -3.0158 | -1.3277 | -0.557 | 0.2133 | 1.917 |

XYP: Xiyanping; RDN: Reduning; TRQ: Tanreqing; YHN: Yanhuning.

# Supplementary File 1 The Detailed Search Strategy

**Pubmed database：**

#1: ((((((((influenza[MeSH Terms]) OR (flu[MeSH Terms])) OR (influenza[Title/Abstract])) OR (flu[Title/Abstract])) OR (seasonal influenza[Title/Abstract])) OR (seasonal flu[Title/Abstract])) OR (flu virus[Title/Abstract])) OR (influenza virus[Title/Abstract])) OR (influenza infections[Title/Abstract])

Results 121543

#2: (((((((((traditional Chinese medicine injection[Title/Abstract]) OR (TCM injection[Title/Abstract])) OR (Tanreqing Injection[Title/Abstract])) OR (Reduning Injection[Title/Abstract])) OR (Xiyanping Injection[Title/Abstract])) OR (Yanhuning Injection[Title/Abstract])) OR (Tanreqing[Title/Abstract])) OR (Reduning[Title/Abstract])) OR (Xiyanping[Title/Abstract])) OR (Yanhuning[Title/Abstract])

Results 295

#3: ((((((((randomized controlled study[Title/Abstract]) OR (randomized controlled trial[Title/Abstract])) OR (randomized trial[Title/Abstract])) OR (randomized study[Title/Abstract])) OR (randomized placebo-controlled study[Title/Abstract])) OR (randomized parallel-group study[Title/Abstract])) OR (randomized placebo controlled[Title/Abstract])) OR (randomized double-blin*[Title/Abstract])) OR (controlled clinical trial[Title/Abstract])

Results 244352

#4：#1 AND #2 AND#3

**Results 2**

**Cochrane library database：**

#1: (flu or seasonal influenza or seasonal flu or influenza or flu virus or influenza virus or influenza infections):ti,ab,kw

Results 10074

#2: (Traditional Chinese Medicine Injection or Tanreqing Injection or Reduning Injection or Xiyanping Injection or Yanhuning Injection or Tanreqing or Reduning or Xiyanping or Yanhuning):ti,ab,kw

Results 488

#3: (randomized controlled study or randomized controlled trial or randomized trial or randomized study or randomized placebo-controlled study or randomized parallel-group study or randomized placebo controlled or randomized double-blin* or controlled clinical trial):ti,ab,kw

Results 1003108

#4：#1 AND #2 AND#3

**Results 10**

**Embase database：**

#1: flu:ab,ti OR 'seasonal influenza':ab,ti OR 'seasonal flu':ab,ti OR influenza:ab,ti OR 'flu virus':ab,ti OR 'influenza virus':ab,ti OR 'influenza infections':ab,ti

Results 139661

#2: 'traditional chinese medicine injection':ab,ti OR 'tanreqing injection':ab,ti OR 'reduning injection':ab,ti OR 'xiyanping injection':ab,ti OR 'yanhuning injection':ab,ti OR tanreqing:ab,ti OR reduning:ab,ti OR xiyanping:ab,ti OR yanhuning:ab,ti

Results 405

#3: 'randomized controlled study':ab,ti OR 'randomized controlled trial':ab,ti OR 'randomized trial':ab,ti OR 'randomized study':ab,ti OR 'randomized placebo-controlled study':ab,ti OR 'randomized parallel-group study':ab,ti OR 'randomized placebo controlled':ab,ti OR 'randomized double-blin*':ab,ti OR 'controlled clinical trial':ab,ti

Results 331065

#:4：#1 AND #2 AND#3

**Results 1**

**Web of Science：**

#1: flu or seasonal influenza or seasonal flu or influenza or flu virus or influenza virus or influenza infections

Results 138325

#2: Traditional Chinese Medicine Injection or Tanreqing Injection or Reduning Injection or Xiyanping Injection or Yanhuning Injection or Tanreqing or Reduning or Xiyanping or Yanhuning

Results 1958

#3: randomized controlled study or randomized controlled trial or randomized trial or randomized study or randomized placebo-controlled study or randomized parallel-group study or randomized placebo controlled or randomized double-blin* or controlled clinical trial

Results 985342

#:4：#1 AND #2 AND#3

**Results 5**

# Supplementary File 2 References of Included Studies

Chen, J. L., Liang, Y. K., Ma, X., Deng, C. Q. (2019). Observation on the Effect of Xiyanping Combined with Oseltamivir Phosphate Capsules in the Treatment of Influenza. Clinical Medicine & Engineering. 26, 811-812.

Chen, X. J. (2012). Observation on the treatment of 60 cases of mild Influenza A with Tanreqing injection combined with oseltamivir. Chinese Journal of Primary Medicine and Pharmacy. 1684-1685.

Chen, X. R., Peng, J. X., An, Z. H. (2014). Efficacy of oseltamivir phosphate combined with Yanhuning in the treatment of influenza virus infection in children. The Journal of Practical Medicine. 30, 4021-4023.

Dong, X. Y., Zhang, Z., Zhu, H. R., Lin, L. Y., Zhang, Y. (2017). Effect of oseltamivir phosphate combined with Yanhuning on immune function and prognosis in children with influenza virus infection. Chinese Journal of Nosocomiology. 27, 2603-2606.

Du, G. L. (2018). Clinical observation of Xiyanping combined with oseltamivir phosphate in the treatment of infantile influenza. Shanxi Medical Journal. 47, 1166-1168.

Fei, F., Liu, J. (2019). Clinical Effect Analysis of Oseltamivir Phosphate Combined with Yanhuning in the Treatment of Children with Viral Influenza. China & Foreign Medical Treatment. 38, 106-108.

Guo, X. R., FGao, X. F. (2012). Effect of Reduining on high body temperature in influenza-like cases. Chinese Journal of Traditional Medical Science and Technology. 19, 370-371.

Guo, X. R., Gao, X. F., Ge, Y. L. (2011). The observation on the effect of combined therapy of oseltamivir and Tanreqing in suspected H1N1 influenza patients. Modern Preventive Medicine. 38, 4089-4090.

Han, J. T. (2011). Clinical observation of Tanreqing injection combined with oseltamivir in the treatment of Influenza A H1N1. Jilin Medical Journal. 32, 1491-1492.

Hu, J., Yu, Y., Ye, N., Li, L. Q., Qiu, W. J., You, X. H., et al. (2011). Observation on Curative Effect of Reduning Injection in the Treatment of Children's Type A H1N1 Influenza Complicated with Pneumonia. Chinese Journal of Practical Pediatrics. 26, 388.

Hu, J. Y., Gao, D. P., Liu, J., Li, N., Liang, Q. J. (2020). Laboratory index evaluation of treatment of infantile influenza virus infection in combination with oseltamivir. Journal of Medical Forum. 41, 61-64.

Huang, J. L., Yang, H. F. (2018). Effect of oseltamivir phosphate combined with Xiyanping injection on influenza A. Chinese Journal of Urban and Rural Enterprise Hygiene. 33, 18-20.

Li, C. Y., Ding, Y., Sun, Y. Y. (2020). The prevention and control effect of oseltamivir and Yanhuning on children's influenza A H1N1 infection. Chinese Journal of Public Health Engineering. 19, 627-629.

Li, G. (2010). Observation ofTanreqing InjectionCombined with Oseltamivir in Influenza A (H1N1). Journal of Emergency in Traditional Chinese Medicine. 19, 1681-1682.

Li, J. P. (2017). Clinical effect of oseltamivir phosphate combined with Yanhuning on influenza virus infection in children. Inner Mongolia Medical Journal. 49, 1344-1345.

Liu, X. (2021). Observation on the therapeutic efficacy of oseltamivir phosphate combined with potassium sodium pehydroandrographolide succinate on influenza. China Medicine and Pharmacy. 11, 90-92.

Luo, R. J. (2021). Efficacy of Tanreqing injection combined with oseltamivir in the treatment of influenza. Inner Mongolia Journal of Traditional Chinese Medicine. 40, 39-40.

Lv, S. S., Wang, L. X. (2019). Clinical efficacy of oseltamivir combined with Reduning injection in the treatment of severe influenza in children. Renowned Doctor. 241.

Qian, J., Xu, J. R., Shi, L. Q. (2011). Efficacy of Tanreqing Injection Combined with Oseltamivir in the Treatment of Type A H1N1 Influenza. Jilin Medical Journal. 32, 266-267.

Tan, J. H. (2018). Analysis of the effect of oseltamivir phosphate combined with Xiyanping in the treatment of children with influenza A. Journal of Chengde Medical University. 35, 215-217.

Tang, C. B., Bu, Y., N. (2010). Treatment of 30 Cases of High Fever Caused by Influenza A H1N1 with Xiyanping and Western Medicine. Chinese Community Doctors. 12, 141.

Wei, X. H. (2018). Analysis of the effect of oseltamivir phosphate combined with Yanhuning on the immune function and prognosis of children with influenza virus infection. Journal of Frontiers of Medicine. 8, 190-191. chi. doi:10.3969/j.issn.2095-1752.2018.36.167.

Xie, Y. (2010). Efficacy of Tanreqing combined with oseltamivir in the treatment of influenza A H1N1. China Modern Doctor. 48, 47+49.

Xu, L., Yu, Y. B., Zhang, G. B. (2010). Reduning injection combined with oseltamivir in the treatment of suspected influenza patients. Modern Journal of Integrated Traditional Chinese and Western Medicine. 19, 3264.

Ye, Y. H., He, X. (2017). Clinical study of Reduning injection combined with oseltamivir phosphate in the treatment of exogenous wind-heat syndrome. Inner Mongolia Journal of Traditional Chinese Medicine. 36, 61-62.

Zhang, C. H. (2015). Clinical observation on treating Pediatric Influenza in the integrative medicine. Clinical Journal of Chinese Medicine. 7, 79-80.

Zhang, Y. W. (2016). Clinical treatment and preliminary evaluation of the effect of influenza in children. World Latest Medicine Information. 16, 265.

Zhao, S. F. (2019). 108 Cases of Influenza Treated by Intravenous Infusion of Reduning Injection. Chinese Baby. 93. chi. doi:10.3969/j.issn.1671-2242.2019.04.086.

Zhong, Q. Y. (2017). Exploration on the curative effect of Tanreqing combined with oseltamivir in the treatment of infantile influenza. World Latest Medicine Information. 17, 92.

Zhong, X. J. (2018). Effect of Yanhuning injection combined with oseltamivir phosphate capsule on immune function in children with viral influenza. Chinese Journal of Clinical Rational Drug Use. 11, 92-93.

Zhu, B. H., Li, Y. X., Xie, H. (2018). Clinical observation on Xiyanping injection in the treatment of children with influenza. Chinese Pediatrics of Integrated Traditional and Western Medicine. 10, 259-261.

# Supplementary PRISMA Checklist

| **Section and Topic** | **Item #** | **Checklist item** | **Location where item is reported** |
| --- | --- | --- | --- |
| **TITLE** | | |  |
| Title | 1 | Identify the report as a systematic review. | 1 |
| **ABSTRACT** | | |  |
| Abstract | 2 | See the PRISMA 2020 for Abstracts checklist. | 2 |
| **INTRODUCTION** | | |  |
| Rationale | 3 | Describe the rationale for the review in the context of existing knowledge. | 4 |
| Objectives | 4 | Provide an explicit statement of the objective(s) or question(s) the review addresses. | 5 |
| **METHODS** | | |  |
| Eligibility criteria | 5 | Specify the inclusion and exclusion criteria for the review and how studies were grouped for the syntheses. | 6 |
| Information sources | 6 | Specify all databases, registers, websites, organisations, reference lists and other sources searched or consulted to identify studies. Specify the date when each source was last searched or consulted. | 6 |
| Search strategy | 7 | Present the full search strategies for all databases, registers and websites, including any filters and limits used. | 6 |
| Selection process | 8 | Specify the methods used to decide whether a study met the inclusion criteria of the review, including how many reviewers screened each record and each report retrieved, whether they worked independently, and if applicable, details of automation tools used in the process. | 6 |
| Data collection process | 9 | Specify the methods used to collect data from reports, including how many reviewers collected data from each report, whether they worked independently, any processes for obtaining or confirming data from study investigators, and if applicable, details of automation tools used in the process. | 7 |
| Data items | 10a | List and define all outcomes for which data were sought. Specify whether all results that were compatible with each outcome domain in each study were sought (e.g. for all measures, time points, analyses), and if not, the methods used to decide which results to collect. | 7 |
|  | 10b | List and define all other variables for which data were sought (e.g. participant and intervention characteristics, funding sources). Describe any assumptions made about any missing or unclear information. | 7 |
| Study risk of bias assessment | 11 | Specify the methods used to assess risk of bias in the included studies, including details of the tool(s) used, how many reviewers assessed each study and whether they worked independently, and if applicable, details of automation tools used in the process. | 7 |
| Effect measures | 12 | Specify for each outcome the effect measure(s) (e.g. risk ratio, mean difference) used in the synthesis or presentation of results. | 7 |
| Synthesis methods | 13a | Describe the processes used to decide which studies were eligible for each synthesis (e.g. tabulating the study intervention characteristics and comparing against the planned groups for each synthesis (item #5)). | 8 |
|  | 13b | Describe any methods required to prepare the data for presentation or synthesis, such as handling of missing summary statistics, or data conversions. | 8 |
|  | 13c | Describe any methods used to tabulate or visually display results of individual studies and syntheses. | 8 |
|  | 13d | Describe any methods used to synthesize results and provide a rationale for the choice(s). If meta-analysis was performed, describe the model(s), method(s) to identify the presence and extent of statistical heterogeneity, and software package(s) used. | 8 |
|  | 13e | Describe any methods used to explore possible causes of heterogeneity among study results (e.g. subgroup analysis, meta-regression). | 8 |
|  | 13f | Describe any sensitivity analyses conducted to assess robustness of the synthesized results. | 8 |
| Reporting bias assessment | 14 | Describe any methods used to assess risk of bias due to missing results in a synthesis (arising from reporting biases). | 7 |
| Certainty assessment | 15 | Describe any methods used to assess certainty (or confidence) in the body of evidence for an outcome. |  |
| **RESULTS** | | |  |
| Study selection | 16a | Describe the results of the search and selection process, from the number of records identified in the search to the number of studies included in the review, ideally using a flow diagram. | 9 |
|  | 16b | Cite studies that might appear to meet the inclusion criteria, but which were excluded, and explain why they were excluded. | 9 |
| Study characteristics | 17 | Cite each included study and present its characteristics. | 9 |
| Risk of bias in studies | 18 | Present assessments of risk of bias for each included study. | 9 |
| Results of individual studies | 19 | For all outcomes, present, for each study: (a) summary statistics for each group (where appropriate) and (b) an effect estimate and its precision (e.g. confidence/credible interval), ideally using structured tables or plots. | 10 |
| Results of syntheses | 20a | For each synthesis, briefly summarise the characteristics and risk of bias among contributing studies. | 10 |
|  | 20b | Present results of all statistical syntheses conducted. If meta-analysis was done, present for each the summary estimate and its precision (e.g. confidence/credible interval) and measures of statistical heterogeneity. If comparing groups, describe the direction of the effect. | 10 |
|  | 20c | Present results of all investigations of possible causes of heterogeneity among study results. | 10 |
|  | 20d | Present results of all sensitivity analyses conducted to assess the robustness of the synthesized results. | 12 |
| Reporting biases | 21 | Present assessments of risk of bias due to missing results (arising from reporting biases) for each synthesis assessed. | 9 |
| Certainty of evidence | 22 | Present assessments of certainty (or confidence) in the body of evidence for each outcome assessed. |  |
| **DISCUSSION** | | |  |
| Discussion | 23a | Provide a general interpretation of the results in the context of other evidence. | 13 |
|  | 23b | Discuss any limitations of the evidence included in the review. | 15 |
|  | 23c | Discuss any limitations of the review processes used. | 15 |
|  | 23d | Discuss implications of the results for practice, policy, and future research. | 14 |
| **OTHER INFORMATION** | | |  |
| Registration and protocol | 24a | Provide registration information for the review, including register name and registration number, or state that the review was not registered. | 5 |
|  | 24b | Indicate where the review protocol can be accessed, or state that a protocol was not prepared. | 5 |
|  | 24c | Describe and explain any amendments to information provided at registration or in the protocol. | 5 |
| Support | 25 | Describe sources of financial or non-financial support for the review, and the role of the funders or sponsors in the review. | 16 |
| Competing interests | 26 | Declare any competing interests of review authors. | 16 |
| Availability of data, code and other materials | 27 | Report which of the following are publicly available and where they can be found: template data collection forms; data extracted from included studies; data used for all analyses; analytic code; any other materials used in the review. | 17 |
